# Visualizing and analyzing 3D biomolecular structures using Mol* at RCSB.org: Influenza A H5N1 virus proteome case study

**DOI:** 10.1002/pro.70093

**Published:** 2025-03-18

**Authors:** Sebastian Bittrich, Alexander S. Rose, David Sehnal, Jose M. Duarte, Yana Rose, Joan Segura, Dennis W. Piehl, Brinda Vallat, Chenghua Shao, Charmi Bhikadiya, Jesse Liang, Mark Ma, David S. Goodsell, Stephen K. Burley, Shuchismita Dutta

**Affiliations:** ^1^ Research Collaboratory for Structural Bioinformatics Protein Data Bank, San Diego Supercomputer Center University of California San Diego La Jolla California USA; ^2^ Mol* Consortium San Diego California USA; ^3^ National Centre for Biomolecular Research, Faculty of Science Masaryk University Brno Czech Republic; ^4^ Research Collaboratory for Structural Bioinformatics Protein Data Bank, Institute for Quantitative Biomedicine, Rutgers The State University of New Jersey Piscataway New Jersey USA; ^5^ Rutgers Cancer Institute, Rutgers The State University of New Jersey New Brunswick New Jersey USA; ^6^ Department of Integrative Structural and Computational Biology The Scripps Research Institute La Jolla California USA; ^7^ Rutgers Artificial Intelligence and Data Science (RAD) Collaboratory, Rutgers The State University of New Jersey Piscataway New Jersey USA; ^8^ Department of Chemistry and Chemical Biology, Rutgers The State University of New Jersey Piscataway New Jersey USA

**Keywords:** 3D biostructure, global health, influenza A H5N1 virus, molecular visualization, open‐source, pandemic preparedness, Protein Data Bank, viral pathogen, virus life cycle, web‐based

## Abstract

The easiest and often most useful way to work with experimentally determined or computationally predicted structures of biomolecules is by viewing their three‐dimensional (3D) shapes using a molecular visualization tool. Mol* was collaboratively developed by RCSB Protein Data Bank (RCSB PDB, RCSB.org) and Protein Data Bank in Europe (PDBe, PDBe.org) as an open‐source, web‐based, 3D visualization software suite for examination and analyses of biostructures. It is capable of displaying atomic coordinates and related experimental data of biomolecular structures together with a variety of annotations, facilitating basic and applied research, training, education, and information dissemination. Across RCSB.org, the RCSB PDB research‐focused web portal, Mol* has been implemented to support single‐mouse‐click atomic‐level visualization of biomolecules (e.g., proteins, nucleic acids, carbohydrates) with bound cofactors, small‐molecule ligands, ions, water molecules, or other macromolecules. RCSB.org Mol* can seamlessly display 3D structures from various sources, allowing structure interrogation, superimposition, and comparison. Using influenza A H5N1 virus as a topical case study of an important pathogen, we exemplify how Mol* has been embedded within various RCSB.org tools—allowing users to view polymer sequence and structure‐based annotations integrated from trusted bioinformatics data resources, assess patterns and trends in groups of structures, and view structures of any size and compositional complexity. In addition to being linked to every experimentally determined biostructure and Computed Structure Model made available at RCSB.org, Standalone Mol* is freely available for visualizing any atomic‐level or multi‐scale biostructure at rcsb.org/3d-view.

## 1 INTRODUCTION

The volume and complexity of three‐dimensional (3D) structure data for biological macromolecules (i.e., proteins, nucleic acids, carbohydrates, lipids, and their various complexes) is growing rapidly. At the time of writing, the Protein Data Bank or PDB (wwPDB consortium 2019) archives and makes available >230,000 3D biostructures, experimentally determined using macromolecular crystallography (MX), 3D electron microscopy (3DEM), or nuclear magnetic resonance (NMR) spectroscopy. In addition, hundreds of millions of computed structure models (CSMs) of proteins are freely available from the AlphaFold Protein Structure Database (Varadi et al. 2024) and the ModelArchive (Humphreys et al. 2021). The easiest way to interact with and explore these structures is to view them using molecular visualization tools. Since PDB was established as the first open‐access digital data resource in biology (Protein Data Bank 1971), the quality of molecular rendering and the capabilities to visualize large and complex data sets have improved dramatically (Li and Wei 2024). Most modern 3D visualization tools offer options for changing representation and analyzing biomolecular structures at the atomic level. Importantly, newer web‐based tools that operate entirely within a user's browser make macromolecular visualization accessible to anyone in the world using a desktop computer, a tablet, or a smartphone connected to the internet.

The Research Collaboratory for Structural Bioinformatics Protein Data Bank (RCSB PDB) (Berman et al. 2000; Burley et al. 2023; Burley et al. 2025) serves as the United States (US) data center of the Worldwide Protein Data Bank (wwPDB, wwpdb.org) partnership, which was established in 2003 to support joint management of the PDB archive as a global public good (Berman et al. 2003). The wwPDB was originally co‐founded by RCSB PDB, Protein Data Bank in Europe (PDBe) (Armstrong et al. 2020), and Protein Data Bank Japan (PDBj) (Bekker et al. 2022). It has expanded over the years to encompass two specialist data resources: Electron Microscopy Data Bank (EMDB) (wwPDB Consortium 2023) and Biological Magnetic Resonance Data Bank (BMRB) (Hoch et al. 2023). Together, RCSB PDB, PDBe, PDBj, EMDB, and BMRB now jointly manage complete deposition (Young et al. 2017), rigorous validation (Baskaran et al. 2024; Feng et al. 2021; Gore et al. 2017; Smart et al. 2018), and expert biocuration (Young et al. 2021) of data contributed to all three wwPDB Core Archives (PDB, EMDB, and BMRB). Recently, PDB China (PDBc) (Xu et al. 2023) joined the wwPDB partnership as an Associate Member.

The RCSB PDB research‐focused RCSB.org web portal provides open‐access tools that support query, browsing, visualization, analysis, comparison, and mapping of annotations for >230,000 experimentally determined 3D biostructures plus more than 1 million CSMs of proteins. Herein, we describe the development of the Mol* web‐based molecular visualization software suite and its deployment within various RCSB.org tools. We exemplify the use of these powerful tools to study the proteome of the influenza A H5N1 virus, a globally important pathogen and a major public health concern recently in the news.

### 1.1 Mol*: An international open‐source software development project

The advent of the Web Graphics Library (WebGL) (khronos.org/webgl/) marked a significant milestone in web application development, enabling robust, high‐performance rendering of 3D visualizations within web browsers. This technical advance eliminated the need for dedicated software installations, such as desktop‐based viewers like PyMOL (DeLano 2002) or ChimeraX (Meng et al. 2023). Two early adopters, the NGL Viewer (developed by RCSB PDB; Rose and Hildebrand 2015) and the LiteMol Suite (developed by PDBe; Sehnal et al. 2017), leveraged their respective capabilities to pioneer the development of more efficient data exchange formats (Bradley et al. 2017; Sehnal et al. 2020). To avoid duplication of effort, these parallel activities were merged with the launch of the open‐source Mol* technology stack (Sehnal et al. 2018; Sehnal et al. 2021) by RCSB PDB, PDBe, and CEITEC (Central European Institute of Technology, Brno, Czech Republic).

Mol* integrated elements of both NGL and LiteMol, establishing a new ecosystem of related data conversion and delivery tools to power 3D visualization of biomolecules (Sehnal et al. 2021). Initially, Mol* was implemented at RCSB.org and PDBe.org, with subsequent adoption by other trusted biodata resources (e.g., UniProt (UniProt Consortium 2023) and AlphaFold Protein Structure Database (Varadi et al. 2024)). Mol* is an excellent choice for an in‐browser biomolecule viewer, offering high‐performance visualization and broad data format support. Other established tools, such as JSmol (Hanson et al. 2013), provide relevant features like extensive scripting capabilities for automating tasks and customizing visualizations—functionality not currently available in Mol*. Additionally, while Mol* excels in visualizing large and complex biomolecular systems, some users may find that it lacks advanced molecular dynamics simulation tools (e.g., VMD; Humphrey et al. 1996).

### 1.2 Mol* capabilities

Mol* supports sophisticated visualization capabilities, rivaling those of traditional desktop applications, while providing a seamless, installation‐free user experience. It can display various representations of atomic coordinates of biomolecular structures, MX electron density maps, 3DEM electric Coulomb potential maps (or density maps), molecular dynamics trajectories, and geometric shapes (e.g., polyhedra) in various formats (Sehnal et al. 2021). In addition, Mol* enables users to analyze and compare structures (e.g., measure interatomic distances, calculate angles, and superimpose structures). Finally, it can save scenes and export images, videos, and sessions for use in publications, presentations, and scientific collaborations. See the Mol* project page (molstar.org/viewer-docs) for detailed instructions. The versatility of Mol* makes it a powerful tool for visualizing biomolecules, enabling dozens of third‐party applications to be built atop the viewer. It has exceptional performance across length scales—supporting visualization of biomolecules ranging from small chemicals to mesoscale atomic models of entire virus capsids or even whole cells (see the Mesoscale Explorer Tool, available at molstar.org/me; Rose et al. 2024).

### 1.3 Integration into the RCSB.org web portal

In 2020, Mol* became the default visualization tool and interaction interface at RCSB.org, supporting access and exploration of 3D biostructure data (hereafter RCSB.org Mol*). It has been linked to RCSB.org Structure Summary Pages (SSPs) across the entire archive, providing single‐mouse‐click access for displaying and analyzing atomic coordinates, macromolecular assemblies, experimental density maps, etc. More recently, Mol* has been deployed within various RCSB.org data display and analysis tools. It is used in the Sequence Annotations Tool (Segura et al. 2020; Segura et al. 2022) to display bidirectional mapping of sequence‐based annotations from trusted bioinformatics data resources on 3D structures. It also powers our Pairwise Structure Alignment Tool (Bittrich et al. 2024b). Mol* can also be used to visualize small‐molecule ligands and biologically interesting molecules (Biologically Interesting molecules Reference Dictionary or BIRD molecules) (Dutta et al. 2014; Sen et al. 2014) within their respective summary pages. Relevant implementations are summarized in Table 1, concerning examples presented below.

**TABLE 1:** Exploring structure and function of influenza A H5N1 virus proteins using RCSB.org Mol* and its deployments within various RCSB.org tools.

H5N1 biology	Protein(s) and/or complex(es)	Focus	Mol* capability	Number
Structures of H5N1 proteins	HA: PDB ID 2fk0 (Stevens et al. 2006)	Overall shape and assembly	Basic Mol* features (Sehnal et al. 2021)	2A, 2B, 2D
		Protein glycosylation patterns	Glycan visualization (Neelamegham et al. 2019; Shao et al. 2021)	2C
		Side‐by‐side, linked display of functional annotations of 1D sequence and 3D structure	Sequence annotations in 1D/3D (Segura et al. 2020; Segura et al. 2022)	3B
		Goodness‐of‐fit of atomic coordinates to experimental density maps	Electron density map visualization	3C
		Structure quality	wwPDB validation reports	3D
			Real‐space correlation coefficient (Shao et al. 2022)	3E
	NA, bound to an inhibitor (zanamivir): PDB ID 3ckz (Collins et al. 2008)	Atomic‐level structures of small‐molecule ligands or BIRD molecules	Ligand and BIRD Summary Pages (Dutta et al. 2014; Sen et al. 2014)	4B, 4E
		Interactions in the vicinity of an amino acid residue or a bound small molecule	Non‐covalent interactions	4C
		Local and global symmetry and pseudosymmetry in biomolecular assemblies	Symmetry visualization	4D
	M2 bound to an inhibitor: PDB ID 6nv1 (Thomaston et al. 2020)	Mapping of phospholipid bilayer location onto membrane protein structures	Predict membrane position (Bittrich et al. 2022)	5B
		Selectively show/hide polypeptide chains, amino acid residues, ligands, and their interactions to explain and illustrate function	Custom representations	5C–5E
	NP: PDB ID 2q06 (Ng et al. 2008); NP bound to RNA: PDB ID 7dxp (Tang et al. 2021)	Compare distinct structures of the same protein to learn about conformational changes (e.g., upon RNA binding)	Standalone Mol* and built‐in alignment tools	6A–6D
Key steps in virus life cycle	Transmissible and non‐transmissible HAs bound to human and avian sialic acid receptors: PDB IDs 4bh0, 4bh1,4bh3, 4bh4 (Xiong et al. 2013)	Compare structures of evolutionarily related proteins interacting with human versus avian sialic acid receptors to understand zoonotic H5N1 infection at the atomic level	Pairwise structure alignment (Bittrich et al. 2024b). Close up and examination of interactions in the neighborhood of specific amino acids in these structures	7A–7F
	H5N1 RNA‐dependent RNA polymerase (RdRp) bound to human ANP32B: PDB ID 8r1j (Staller et al. 2024)	Overview of structure and visualization of location of key components of the complex	Transparent surface	8A
		Launch views of all structures including any part of ANP32B annotated with the same UniProt ID to compare the PDB structure of ANP32B with that of its AlphaFold2 CSM	Sequence Alignment Viewer (Segura et al. 2020; Segura et al. 2022)	8C
	AlphaFold2 CSM of human ANP32B: RCSB.org ID AF_AFQ92688F1 (Jumper et al. 2021)	View CSM color coded by prediction confidence (dark blue denotes highest confidence)	pLDDT coloring	8D
		Side‐by‐side display of structural and functional annotations on both 1D sequence and 3D structure	Sequence Annotations Viewer	8E
Structure exploration facilitating antiviral drug discovery	Comparison of NA co‐crystal structures with zanamivir and oseltamivir carboxylate: PDB IDs 3ckz, 3cl0 (Collins et al. 2008)	Identify common interactions for drug binding	Sequence Alignment View (Segura et al. 2020; Segura et al. 2022)	9A, 9B
	Select NA amino acid residues interacting with zanamivir for Structure Motif Search	Identify 3D biostructures (experimentally determined or computationally predicted) at RCSB.org with the defined structure motif (for possible zanamivir binding sites in human sialidases, etc.)	Structure Motif Search (Bittrich et al. 2020)	9C–9F

*Note*: Core Mol* features have been described previously (Sehnal et al. 2021).

### 1.4 Introducing the Influenza A H5N1 virus case study

Influenza infections (the “flu”) are caused by influenza viruses. Reports of flu epidemics affecting humans date as far back as the 5th century BCE (Potter 2001). One of the worst pandemics recorded in the 20th century was the 1918 Spanish flu, which killed an estimated 50 million people worldwide (Taubenberger and Morens 2009). All influenza viruses are enveloped, negative‐sense, single‐stranded RNA viruses with segmented genomes (Taubenberger and Morens 2010). Among the four known types of influenza virus (A, B, C, D), subtypes of A have caused pandemics in the modern era (Centers for Disease Control and Prevention and National Center for Immunization and Respiratory Diseases (NCIRD) 2023). Two viral envelope proteins, hemagglutinin (HA) and neuraminidase (NA), encoded by distinct genome segments, vary among influenza A virus subtypes. Combinations of 18 different HAs (HA1 through HA18) and 11 different NAs (NA1 through NA11) can yield many different viruses identified with H#N# designations (Centers for Disease Control and Prevention and National Center for Immunization and Respiratory Diseases (NCIRD) 2023). New combinations of HA and NA can arise when host cells become co‐infected by two or more viruses with distinct HAs and/or NAs.

Some influenza A viruses can jump the species barrier from one host to another, for example, from birds or pigs to humans (World Health Organization 2023). While such zoonotic infections rarely spread from person to person, aquatic birds serve as a vast natural for influenza A viruses. Exposure to infected birds (dead or alive) can constitute a substantial public health risk. From 1997, when the first case of a human infected with an influenza A H5N1 virus was detected, to April 2024, 912 sporadic cases of human influenza A H5N1 virus infections have been reported in 24 countries with disturbingly high case‐fatality rates exceeding 50% (Centers for Disease Control and Prevention 2024). More recently, so‐called “bird flu” viruses have been shown to infect dairy cows (Abbasi 2024). Hence, dairy workers are now thought to be at higher risk of infection than the general population (Garg et al. 2024), raising concerns about the possibility of a serious epidemic or even a global pandemic that could kill millions.

**FIGURE 1:**
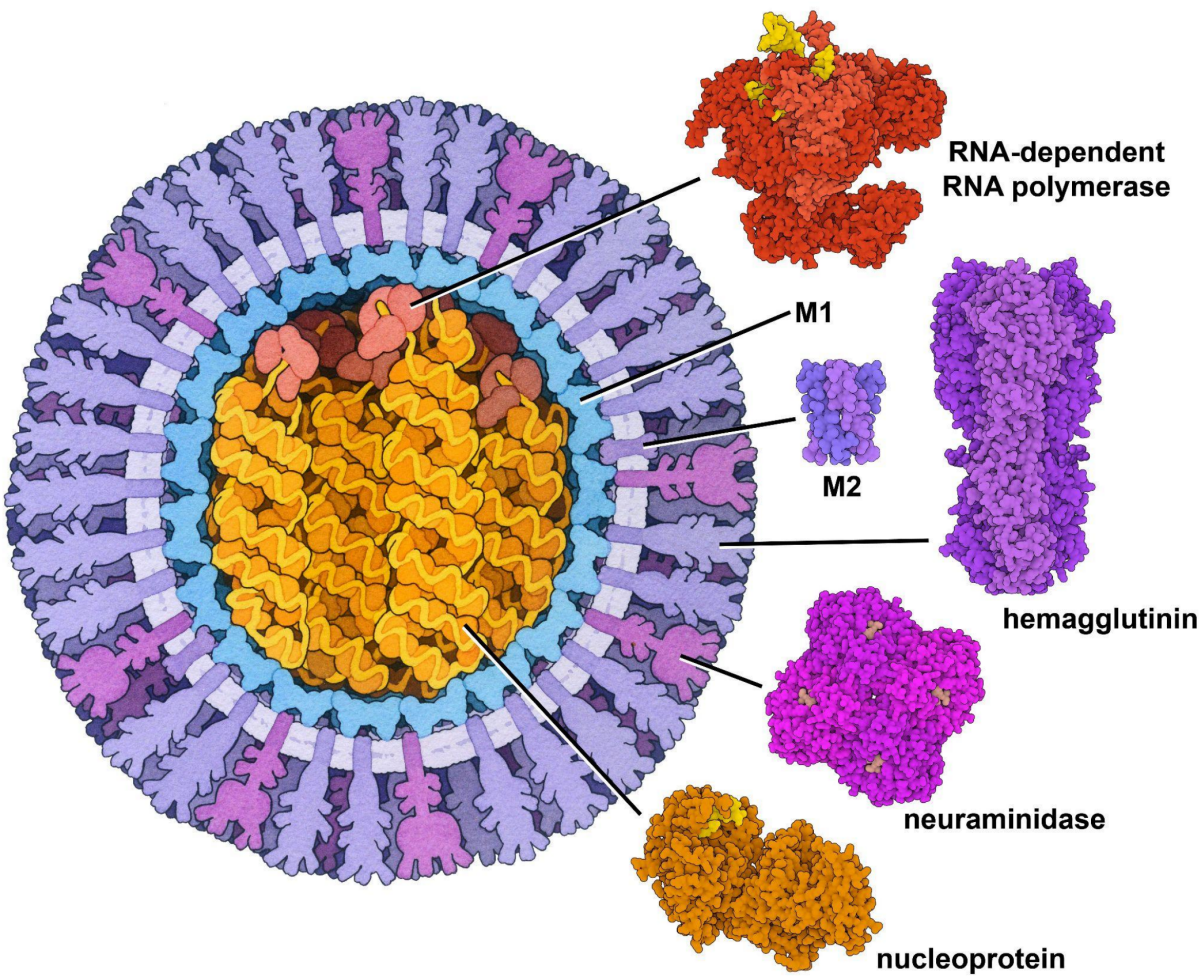
David Goodsell's rendition of the H5N1 virion in cross‐section (left) and experimentally determined structures of select H5N1 proteins (right) drawn using Mol*: RdRp (PDB ID 8h69; Li et al. 2023), M2 (PDB ID 6nv1; Thomaston et al. 2020), HA (PDB ID 2fk0; Stevens et al. 2006), NA (PDB ID 2hu4; Russell et al. 2006), and NP (PDB ID 7dxp; Tang et al. 2021). The shape of M1 in the artistic rendition is based on structures from other influenza A viruses (e.g., PDB ID 7jm3; Selzer et al. 2020).

To exemplify the utility of RCSB.org Mol* and RCSB.org tools reliant on Mol*, we present influenza A H5N1 virus (hereafter H5N1) as a topical case study (Figure 1). We use data from the PDB archive and trusted bioinformatics resources to explore the H5N1 proteome in 3D at the atomic level, to examine key steps in the influenza A virus life cycle, and discuss the potential impact of structural knowledge of the H5N1 proteome for epidemic/pandemic preparedness (i.e., discovering/developing drug therapies or designing vaccines to counter H5N1). Distinct deployments of Mol* used in these explorations are summarized in Table 1, with reference to examples presented herein. Instructions for recreating Figure 1 using Mol* are provided in Figure S1, Supporting Information, with similar details for subsequent figures in their corresponding figures.

## 2 RESULTS

### 2.1 Architecture of the H5N1 virion

Figure 1 (left) presents an artist's depiction of a cross‐sectional view of the H5N1 virion (Goodsell 2024) that integrates 3D structure information from MX and 3DEM. The RNA genome (yellow) is shown packaged/protected by many copies of the nucleoprotein (NP, orange), surrounded by a shell of matrix proteins (M1, light blue), and encased within a lipid envelope (mauve). Three distinct populations of surface proteins embedded within the lipid bilayer viral envelope include hemagglutinin (HA, purple), neuraminidase (NA, magenta), and a proton‐conducting channel (M2, purple). Once the virus enters a host cell nucleus, the RNA‐dependent RNA polymerase (RdRp, light red) transcribes viral mRNAs and replicates the viral genome (Du et al. 2023). Experimentally determined 3D structures of functionally important parts of most H5N1 influenza virus proteins are archived in PDB and illustrated using RCSB.org Mol* (Figure 1, right). Hereafter, we exemplify the use of RCSB.org Mol* based tools to view H5N1 protein structures and explore their function.

### 2.2 Exploring the H5N1 proteome in 3D

#### 2.2.1 Hemagglutinin

Hemagglutinin (HA) is the major viral surface protein. It is a homotrimeric glycoprotein in the influenza virus envelope that recognizes sialic acid‐containing glycans displayed on host‐cell surfaces to gain viral entry. The protein is synthesized as a long single polypeptide chain (HA0). Late in the infection, host cell proteases cleave HA0 into two segments (HA1, the sialic acid receptor binding subunit; HA2, the membrane fusion subunit), which are covalently linked to one another by disulfide bridges (Antanasijevic et al. 2020). At the time of writing, PDB holdings included >500 experimentally determined structures of influenza A virus HAs.

**FIGURE 2:**
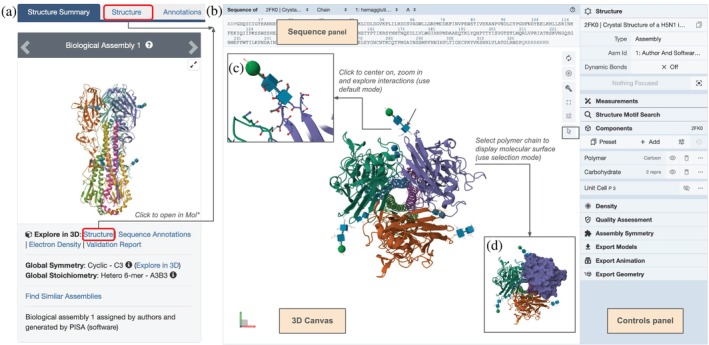
Structure of H5N1 HA trimer (PDB ID 2fk0; Stevens et al. 2006). (a) Screenshot of the top left corner of the SSP. (b) Single‐mouse‐click of the Structure tab or the hyperlinked word Structure (red boxes in (a)) launches RCSB.org Mol*, revealing Sequence panel, 3D Canvas, and Controls panel. (c) Close‐up view of a covalently‐bound glycan attached to Asn169, illustrated using Symbol Nomenclature for Glycans representation (Neelamegham et al. 2019; Shao et al. 2021), showing nearby covalent and non‐covalent interactions. (d) View of the symmetric homotrimer with one HA depicted with molecular surface representation (invoked in Mol* Selection mode).

The first H5N1 HA structure contributed to PDB was determined using MX at ~2.95 Å resolution (PDB ID 2fk0) (Stevens et al. 2006). A snapshot of the homotrimeric structure is displayed in the top left corner of its SSP (Figure 2a). The image consists of three copies of each of the HA1 (green, orange, and purple) and HA2 (yellow, light green, and pink) protein chains. Overall, the stalk‐like hemagglutinin trimer has a bulbous head with three receptor binding sites for binding sialic acid moieties occurring within host‐cell surface glycoprotein glycans. All HAs are glycosylated. Blue cubes and green spheres linked to the polypeptide chains shown throughout Figure 2 denote experimentally observed carbohydrate post‐translational modifications.

Single‐mouse‐click of the Structure tab or the hyperlinked Structure button at the bottom of the thumbnail image shown in the top left corner of the SSP (Figure 2a) launches RCSB.org Mol*, revealing the 3D structure at the atomic level (Figure 2b). It utilizes (i) a Sequence panel that lists polymer sequences and small molecule entities present in the structure; (ii) a 3D Canvas that displays the biomolecule structure interactively; and (iii) a Controls panel that provides options for managing the graphical display. RCSB.org Mol* operates in two different modes. In default mode, clicking on any part of the structure positions it at the center of the 3D Canvas, zooms in, and displays both covalent and non‐covalent interactions within a 5 Å radius. The outcome of a single‐mouse‐click on the glycan marked with a black arrow is shown in Figure 2c. The Mol* Selection mode is activated by clicking on the open arrow button in the vertical menu on the right side of the 3D Canvas. Doing so presents a horizontal menu at the top of the 3D Canvas with options to select and change representations and colors of the selected portion of the 3D biostructure. Figure 2d shows the HA protein representation changed to a molecular surface representation for one protomer within the symmetric homotrimer (see Figure S2 for details).

**FIGURE 3:**
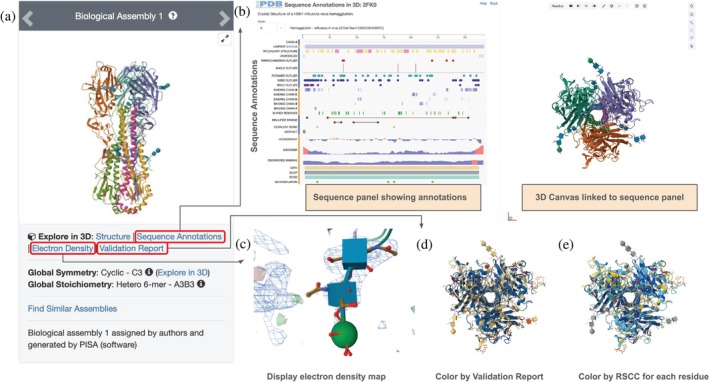
Exploring the H5N1 HA homotrimer (PDB ID 2fk0; Stevens et al. 2006). (a) Screenshot of the top left corner of the SSP. (b) Interface showing Sequence Annotations mapped onto the 3D structure in the linked side‐by‐side view. (c) Electron density map for a selected portion of a monomer structure. (d) Ribbon representation of the HA homotrimer colored by wwPDB Validation Report issues. (e) Same HA homotrimer colored by individual residue RSCC values.

Every SSP offers various single‐mouse‐click features providing immediate access to different representations and aspects of structure. The Sequence Annotations button visible in Figure 3a presents the 1D polymer sequence and 3D biostructure in side‐by‐side linked panels (Figure 3b). Clicking on the Electron Density button (Figure 3a) shows available experimental density maps (Figure 3c). Clicking on the Validation Report button (Figure 3a) displays the entire structure, colored based on structure quality—parts of the structure with no or few wwPDB validation‐reported issues are shown in blue, while regions with more issues are shown in orange and yellow (Figure 3d). Although not directly available from the SSP, an additional validation color scheme for MX structures identifies per‐residue confidence based on the real‐space correlation coefficient (RSCC) of experimentally determined electron density versus atomic coordinates (Shao et al. 2022) (Figure 3e, available from the Quality Assessment section of the controls panel in RCSB.org Mol*). See Figure S3 for details.

#### 2.2.2 Neuraminidase

Neuraminidase (NA) is the second major influenza virus surface glycoprotein. It is an enzyme that acts on glycans to cleave terminal sialic acid residues (from both viral and host cell glycoproteins). The enzymatic activity of NA is essential for the efficient release of newly assembled virions from host cells because it prevents them from binding and being endocytosed. At the time of writing, PDB holdings included >200 experimentally determined structures of influenza A virus NAs.

**FIGURE 4:**
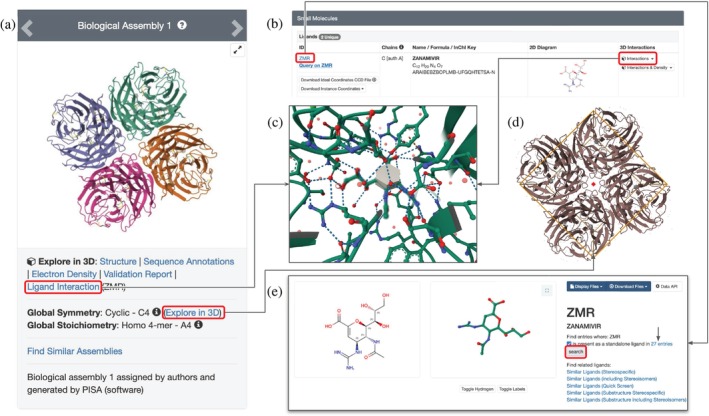
Co‐crystal structure of H5N1 NA inhibited by zanamivir (PDB ID 3ckz; Collins et al. 2008). (a) Screenshot of the top left corner of the SSP. (b) Exploring covalent and non‐covalent interactions in the vicinity of the bound drug. (c) Screenshot of the Small Molecules section of the SSP. (d) Screenshot of the Ligand Summary Page for zanamivir (ZMR), showing 2D and 3D images of the drug. (e) Exploring the global symmetry of the structure with the C4 symmetry axis denoted by a red square.

Many of the NA structures in PDB provide atomic‐level insight into binding of inhibitors within the enzyme active site. One such inhibitor is a commonly used small‐molecule drug treatment for influenza virus infections—zanamivir (brand name Relenza, wwPDB Chemical Component Dictionary, or CCD ID ZMR). The first PDB structure of an H5N1 NA‐zanamivir complex was determined using MX at 1.9 Å resolution (PDB ID 3ckz) (Collins et al. 2008). The enzyme is a symmetric homotetramer (Figure 4a) of a largely *β*‐sheet protein stabilized by several disulfide bridges. The active site of each protomer in PDB ID 3ckz is occupied by zanamivir. Drug–protein interactions can be viewed with RCSB.org Mol* by single‐mouse‐click of the Ligand Interaction button at the top left corner of the SSP (Figure 4a), or the Interactions button in the Small Molecules section of the SSP (Figure 4b), or by single‐mouse‐click of the bound ligand displayed in the 3D Canvas (in default mode). The resulting zoomed‐in view is centered on zanamivir. All amino acid residues located within 5 Å of the ligand are shown in ball‐and‐stick representation together with nearby non‐covalent intra‐ and inter‐molecular interactions (Figure 4c, blue dashed lines denote hydrogen bonds or H‐bonds, orange dashed lines represent *π*–cation interactions).

Single‐mouse‐click of a button in the top left corner of the SSP (Figure 4a) supports exploration of Global Symmetry in a structure (Duarte et al. 2022). In Figure 4d, the symmetry of the tetrameric NA assembly is denoted by the small red square positioned at the tip of the symmetry axis and a cube‐shaped cage denoting Cyclic Global C4 Symmetry. Clicking the CCD ID in the Small Molecules section of the SSP (Figure 4b) opens the corresponding Ligand Summary Page (Figure 4e), wherein Mol* is used to display the 3D structure of the ligand interactively. Single‐mouse‐click of the Search button in this panel executes a search for all PDB structures containing this ligand. At the time of writing, this search returned 27 hits, including structures of 24 influenza NAs, one human sialidase (PDB ID 2f0z) (Chavas et al. 2005), and two bacterial proteins. See Figure S4 for details.

#### 2.2.3 M2 ion channel

The M2 protein forms an ion channel embedded in the viral envelope. It is necessary for the release of the viral genome into the cytoplasm of the host cell at the beginning of the infectious process and later during virion packaging (Pinto and Lamb 2006). Once the virion is internalized by host cell endocytosis, the resulting endosome is acidified, and the viral membrane fuses with the endosome lipid bilayer. In addition to stabilizing the fusion‐promoting conformation of HA (Skehel and Wiley 2000), the pH drop activates the M2 ion channel, allowing protons to enter the virion nucleocapsid. Doing so disrupts interactions between the viral ribonucleoprotein (RNP), the matrix protein 1 (M1), and the viral membrane, thereby uncoating and ejecting the viral genome into the host cell cytoplasm (Pielak and Chou 2011). During virus maturation in the normally acidic trans‐Golgi network, the M2 ion channel acts locally to increase pH, thereby preventing newly translated HA proteins from assuming the fusion‐promoting conformation.

**FIGURE 5:**
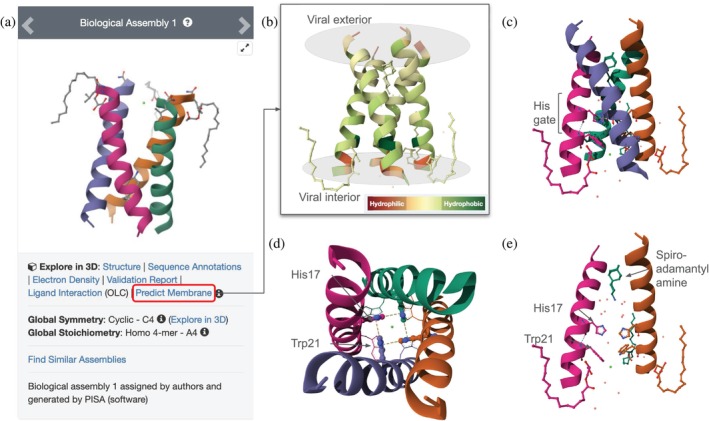
Structure of the H5N1 M2 ion channel pore. (a) Screenshot of the top left corner of the SSP for PDB ID 6nv1 (Thomaston et al. 2020) showing the Predict Membrane button within a red rectangle. (b) RCSB.org Mol* image showing the predicted membrane orientation for PDB ID 6nv1 as circular gray planes corresponding to outer and inner membrane leaflets. Polypeptide chain segments of M2 are shown in ribbon representation, color‐coded for hydrophobicity versus hydrophilicity (see panel inset). (c) Side‐view of the M2 ion channel shown in ribbon representation with different colored polymer chains (orientation as in (b), location of His17 [auth 37] ion gate is marked). (d) Outside‐to‐inside view of the M2 ion focusing on key residues forming the ion gate (histidine residues in ball‐and‐stick representation, tryptophan residues shown as thin lines). (e) Identical view of the M2 ion channel as in panel (c), with two of the polymer chains hidden for clarity showing His17 [auth 37] and Trp21 [auth 41] and the channel blocking ligand.

The M2 ion channel is a 27‐amino acid protein with a transmembrane *α*‐helix that forms a symmetric homotetramer (PDB ID 6nv1) (Thomaston et al. 2020). At the time of writing, PDB archival holdings included >20 experimentally determined structures of influenza A virus M2 proteins. Much of our understanding of M2 function comes from MX studies of hydrophobic synthetic peptides, corresponding to the transmembrane portion of the ion channel (Figure 5a,b), reconstituted in the presence of membrane phospholipid‐mimicking detergents plus spiro‐adamantyl amine (a small‐molecule channel blocker). Single‐mouse‐click of the Predict Membrane button in the top left corner of the SSP (Figure 5a) displays the structure depicted in Figure 5b with two circular planes in the RCSB.org Mol* 3D Canvas, representing the predicted extent of the phospholipid bilayer.

Two amino acid sidechains (His17 [auth 37] and Trp21 [auth 41]), originate from each of the four *α*‐helices pointing towards the center of the ion channel (Note: In some PDB structures, an amino acid residue may possess two identifying numbers: the PDB‐assigned ordinal number (label_seq_id) and an author‐provided number (auth_seq_id)). The sidechains of His17 [auth 37] interact with each other (via *π*–*π* interactions) and water molecules (via H‐bonds) to stabilize the tetrameric assembly and form a gate preventing H^+^ ion flow across the viral membrane. RCSB.org Mol* provides various options to select or change representations, hide or show parts of a structure, and display specific interactions to illustrate a molecular story. Key elements of the M2 gate are shown in side‐ (Figure 5c), top‐ (Figure 5d), and cut‐away side‐views (Figure 5e). Within the acidified endosome (pH ~5.5), the imidazole moiety of His17 [auth 37] is protonated, destabilizing interactions within the M2 gate and allowing passage of protons into the nucleocapsid. Single‐mouse‐click of the His residue (in default mode) displays interactions within 5 Å. See Figure S5 for details.

#### 2.2.4 Nucleoprotein

The H5N1 nucleoprotein (NP) is responsible for packaging the segmented RNA genome within the nucleocapsid. In addition to protecting against nuclease digestion, NP interacts with the viral RNA‐dependent RNA polymerase (RdRp) and plays a role in determining whether the viral genome is transcribed into mRNAs encoding viral proteins or used as a template for genome replication (Ng et al. 2008). Multiple NPs bound to the RNA genome (viral ribonucleoprotein or vRNP) become localized within the host cell nucleus early in the infection process. A bipartite nuclear localization signal (NLS) found within the NP facilitates active nuclear import of vRNPs via binding to karyopherins (Ozawa et al. 2007). Later in the infectious process, this polypeptide chain segment becomes occluded by the matrix protein M1 to prevent inappropriate reimportation of newly assembled vRNPs exported from the nucleus.

**FIGURE 6:**
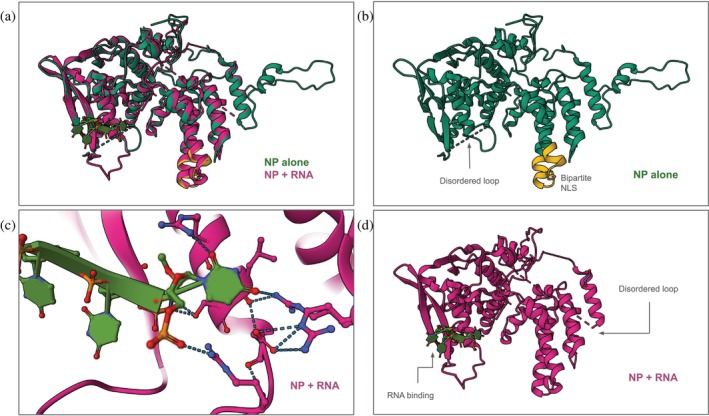
NP conformational changes upon RNA binding. (a) Superimposition of the structures of NP alone (PDB ID 2q06; green; Ng et al. 2008) and NP bound to RNA (PDB ID 7dxp; magenta; Tang et al. 2021). (b) Structure of NP alone, showing the yellow‐colored bipartite NLS (residues 204 [auth 198] to 222 [auth 216]). (c) Zoomed in view of an RNA oligonucleotide bound to the NP showing intermolecular interactions in atomic detail. (d) Structure of NP bound to RNA.

To examine the consequences of conformational changes due to RNA binding, NP structures with and without bound RNA can be superimposed using the RCSB PDB Standalone Mol* (rcsb.org/3d-view; also accessible from the RCSB.org homepage via the “Visualize” dropdown menu). This version of Mol* is not connected to any specific SSP. It supports visualization of multiple experimentally determined PDB structures and/or predicted CSMs and/or uploaded atomic coordinates. Figure 6a shows a ~3.3 Å resolution MX structure of the NP alone (PDB ID 2q06) (Ng et al. 2008) superimposed on a ~2.3 Å resolution MX structure of NP bound to RNA (PDB ID 7dxp) (Tang et al. 2021) generated by Standalone Mol*. Viewing either structure reveals that the NP is an *α*/*β*‐protein (Figure 6b) with a solvent‐accessible bipartite NLS (yellow in Figure 6b). Binding of an RNA oligonucleotide causes loops near the RNA binding site to become ordered (Figure 6c), while loops near the NLS become disordered (compare Figure 6b,d). See sect. 6 of Data S1 for details.

#### 2.2.5 M1 matrix protein

The M1 matrix protein is ~250 amino acids in length. It interacts with both the viral envelope and the segmented RNA genome to form the protein coat of the nucleocapsid. At the time of writing, PDB holdings included ~20 experimentally determined structures of influenza A virus M1 proteins, although none were derived from an H5N1 virus. A ~3.4 Å resolution 3DEM structure of the M1 protein (PDB ID 7jm3; EMDB ID EMD‐22384) (Selzer et al. 2020) revealed that the N‐ and C‐terminal domains are largely *α*‐helical and connected by a flexible linker. RCSB.org Mol* tools exemplified in Figures 2 and 3 can be used to visualize and analyze this protein.

#### 2.2.6 RNA‐dependent RNA polymerase

The viral RdRp is tripartite, consisting of polymerase acidic protein (PA), RdRp catalytic subunit (PB1), and polymerase basic protein 2 (PB2). At the time of writing, PDB holdings included >50 experimentally determined structures of influenza A virus RdRp heterotrimers. The first structure of an H5N1 RdRp heterotrimer was determined via 3DEM at ~3.2 Å resolution (PDB ID 8r1j; EMDB ID EMD‐18818) (Staller et al. 2024). Again, RCSB.org Mol* tools exemplified in Figures 2 and 3 can be used to visualize and analyze this complex assembly.

#### 2.2.7 Influenza A virus life cycle

The influenza A virus life cycle spans binding to sialylated cell‐surface proteins, endocytosis, acidification of the endosome, fusion of the viral lipid bilayer with the endosomal membrane, ejection of the viral nucleocapsid into the host cell cytoplasm, transport of the segmented viral genome into the nucleus, viral RNA transcription and translation, genome replication, and assembly of new virions. Viral transmembrane proteins are translocated into the endoplasmic reticulum (ER) and trafficked to the Golgi, where they induce budding. Cytoplasmic M1 packages the viral genome into budding virions. Finally, sialic acid cleavage by NAs facilitates the release of nascent virions (Carter and Iqbal 2024) ready to infect nearby cells or other animal or human hosts. To further exemplify how RCSB.org Mol* has been deployed within various RCSB PDB structure display and analysis tools, two key steps of the viral life cycle are examined in 3D: (i) species‐specific HA binding and (ii) RdRp switching between mRNA transcription and genome replication.

#### 2.2.8 HA binding to avian versus human sialic acid receptors

**FIGURE 7:**
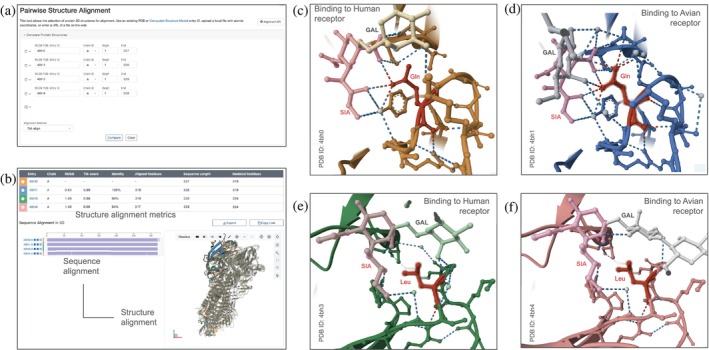
Comparing co‐crystal structures of non‐transmissible (Asn/Gln) and transmissible‐mutant (Lys/Leu) HAs (Xiong et al. 2013). (a) Pairwise Structure Alignment Tool displaying the list of structures to be compared. (b) Results of the above structure comparison (structures are distinctly colored). (c) Non‐transmissible HA (Asn/Gln) bound to a human sialic acid receptor (PDB ID 4bh0). (d) Non‐transmissible HA (Asn/Gln) bound to an avian sialic acid receptor (PDB ID 4bh1). (e) Transmissible‐mutant HA (Lys/Leu) bound to a human sialic acid receptor (PDB ID 4bh3). (f) Transmissible‐mutant HA (Lys/Leu) bound to an avian sialic acid receptor (PDB ID 4bh4).

HA homotrimers displayed on the surface of the viral envelope recognize and bind to sialic acid receptors decorating host cell surface glycoproteins and glycolipids (Figure 7). Bird (or avian) host cells display glycans with sialic acid moieties α2,3‐linked to galactose, whereas glycans in human cells utilize α2,6‐linkages (Shinya and Kawaoka 2006). Thus, for H5N1 to cross the species barrier from birds to humans, changes in HA sialic acid receptor binding preferences must play a role.

The RCSB PDB Pairwise Structure Alignment Tool (Figure 7a; rcsb.org/alignment; also accessible from the RCSB.org homepage via the “Analyze” dropdown menu; Bittrich et al. 2024b) was used to superimpose co‐crystal structures of HAs from two H5N1 virions: non‐transmissible (turkey/Turkey/1/2/005(H5N1): PDB IDs 4bh0—human‐type sialic acid receptor; 4bh1—avian‐type sialic acid receptor) and transmissible/mutant (A/Vietnam/1203/2004(H5N1): PDB IDs 4bh3—human‐type sialic acid receptor; 4bh4—avian‐type sialic acid receptor) (Xiong et al. 2013). The default TM‐align option was used, which leverages secondary structure information to perform a pairwise structure alignment (Zhang and Skolnick 2005). Structure superimposition confirmed that the two proteins are highly similar in 3D structure with root‐mean‐square‐deviation or RMSD ~1.5 Å (PDB IDs 4bh3 vs. 4bh0, sequence identity ~94%, 319 equivalent *α*‐carbon atomic pairs) (Figure 7a,b). Within the sialic acid receptor binding site of transmissible/mutant A/Vietnam/1203/2004(H5N1) HA, two amino acid differences are thought to be important for transmissibility—i.e., Asn 220 is changed to Lys and Gln 222 is changed to Leu (Note: The Xiong et al. 2013 manuscript refers to these amino acids as Asn 224 and Gln 226, respectively. The Pairwise Structure Alignment Tool refers to these amino acids by their assigned ordinal numbers (Asn 222 and Gln 224). However, the paragraph below uses the author‐specified numbering (Asn 220 and Gln 222)).

Figure 7c–f depicts the binding of non‐transmissible HA (Asn/Gln) and transmissible HA (Lys/Leu) to human‐ and avian‐type sialic acid receptors. HA (Asn/Gln) makes several H‐bonds involving Gln 222 and Tyr 91 sidechains with the human‐type sialic acid receptor (PDB ID 4bh0; Figure 7c). When binding to the avian‐type sialic acid receptor, Gln 222 in HA (Asn/Gln) makes similar interactions to those depicted in Figure 7c, plus it forms H‐bonds to a nearby galactose moiety and Ser 133 (PDB ID 4bh1; Figure 7d). These additional interactions enable HA (Asn/Gln) to bind more tightly and selectively to the avian sialic acid receptor than to its human counterpart and may explain non‐transmissibility. Because the Asn 220 sidechain is oriented away from the sialic acid, it has a limited impact on receptor binding.

In contrast, transmissible‐mutant HA (Lys/Leu) makes very few H‐bonds with either type of bound glycan (PDB ID 4bh3—human‐type sialic acid receptor, Figure 7e; PDB ID 4bh4—avian‐type sialic acid receptor, Figure 7f). In both cases, the hairpin conformation of the bound glycan wrapping around the hydrophobic Leu side chain is markedly different from that observed for either of the HA (Asn/Gln) co‐crystal structures shown in Figure 7c,d. Lys 220 forms H‐bonds with backbone atoms of Ala 134 and Cys 90, in both human‐type and avian‐type receptor‐bound structures. Additional water‐mediated H‐bonds are seen when HA (Lys/Leu) binds to the human‐type sialic acid receptor. Biophysical characterization of HA–receptor interactions (Xiong et al. 2013, fig. 1a) documented that HA (Lys/Leu) binds human‐type sialic acid receptors (Figure 7e) more tightly than avian‐type sialic acid receptors (Figure 7f), thereby facilitating infection of both human and avian hosts. See Figure S7 for details.

More recently, Wilson and co‐workers reported the experimental structure determination of an HA from the first‐reported human‐infecting bovine H5N1 virus (A/Texas/37/2024, Texas) using MX at ~2.7 Å resolution (PDB ID 9diq) (Lin et al. 2024). Additional structural studies from Wilson's research group revealed (i) avian‐type sialic acid receptor binding preference (PDbB ID 9dip), and (ii) human‐type sialic acid receptor binding preference for the Gln226Leu modified form of the “Texas HA” (PDB ID 9dio). Moreover, binding to human‐type sialic acid receptors was enhanced for the Gln226Leu/Asn224Lys doubly modified form of the “Texas HA.”

#### 2.2.9 Viral transcription and replication

Once inside the host cell, the segmented viral genome remains bound to one or more NPs, forming viral ribonucleoproteins (vRNPs). The NP NLS mediates the transport of vRNPs and associated RdRps into the nucleus, wherein they both synthesize viral protein mRNAs (transcription) and replicate the segmented viral genome. Primers for transcription initiation are obtained by the RdRp using a process known as “cap snatching” (Plotch et al. 1981). Resulting viral mRNAs are translated by host cell ribosomes, yielding the accumulation of the RdRp, NP, M1, and NP proteins in the cytoplasm, and the HA, NA, and M2 proteins embedded in the lipid bilayer of the ER.

**FIGURE 8:**
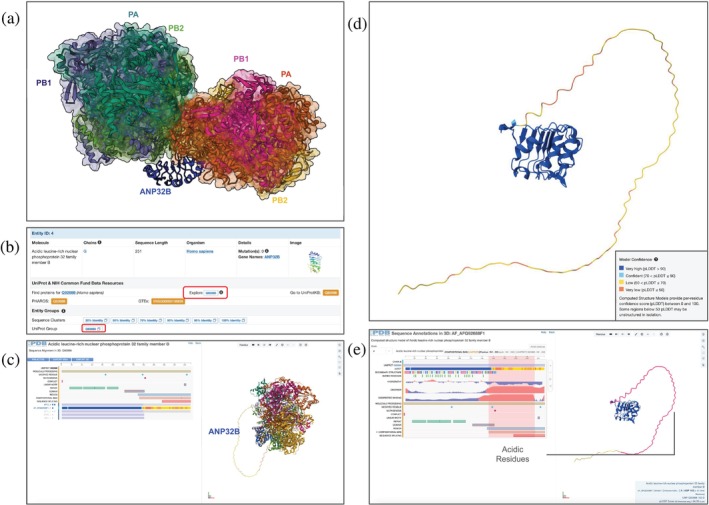
3DEM structure of two copies of H5N1 RdRp bound to one copy of human ANP32B (PDB ID 8r1j; Staller et al. 2024). (a) Component proteins (PA, PB1, and PB2) are shown with semi‐transparent molecular surfaces, bound to ANP32B (dark blue, ribbon representation). (b) Macromolecules section of the SSP with information and links for the human ANP32B protein. (c) Sequence Alignment view for human ANP32B with the amino acid sequence of UniProt Accession Number Q92688. (d) Mol* view of CSM AF_AFQ92688F1, shown in ribbon representation, colored by pLDDT values. (e) Sequence Annotations in 3D showing linked 1D sequence panel (left) and 3D structure display (right).

Replication of the influenza A viral genome in human cells requires one or the other of two related acidic nuclear phosphoproteins (ANP32 A or B) (Staller et al. 2019). The N‐terminal segments of ANP32 A or B contain several leucine‐rich repeats, while their C‐terminal portions include many negatively charged amino acids thought to interact with positively charged viral NPs. A ~3.2 Å resolution 3DEM structure of the H5N1 RdRp heterotrimer (PA, PB1, and PB2) bound to human ANP32B (PDB ID 8r1j) (Staller et al. 2024) is depicted in Figure 8a. The PA subunit interacts with the globular N‐terminal domain of ANP32B (Figure 8a) (Note: Atomic coordinates for the C‐terminal domain of ANP32B are absent in PDB ID 8r1j).

To learn more about the missing C‐terminal portion of ANP32B, RCSB.org can be queried for other structures that may include this region. A single‐mouse‐click of the UniProt Group button from the SSP for PDB ID 8r1j (Figure 8b) opens a Group Summary Page (Segura et al. 2023) displaying information about 3D biostructures, including UniProt Accession Number Q92688 available at RCSB.org. Visualization of the CSM for the full‐length protein (AF_AFQ92688F1, predicted using AlphaFold2) (Jumper et al. 2021) reveals that the C‐terminal portion of the human protein (Figure 8d,e) is likely to be intrinsically disordered (at least in the absence of a binding partner). Inspection of the Sequence Alignment in 3D page (Figure 8c) allows selective viewing of the CSM pre‐aligned with each experimentally determined PDB structure of ANP32B. The polypeptide chain of the CSM is colored (by default) based on per‐residue prediction confidence (predicted local distance difference test or pLDDT), ranging from 0 (lowest confidence, orange) to 100 (highest confidence, dark blue) (Tunyasuvunakool et al. 2021). Viewing Sequence Annotations for ANP32B reveals that its C‐terminal region is rich in acidic residues (Figure 8e), which explains why it is predicted to be unstructured in isolation. See Figure S8 for details.

### 2.3 Preparing for a possible H5N1 epidemic/pandemic

Seasonal influenza viruses cause acute respiratory infections, some of which may require hospitalization and, in rare cases, prove fatal. Zoonotic influenza viruses (causing bird flu, swine flu, etc.) can spread to humans, potentially causing a global public health crisis. Enhanced surveillance, vaccination programs, non‐pharmacologic precautions (e.g., frequent hand washing, N95/KN95 mask usage), and timely clinical management with antiviral agents are proven means of supporting public health and preventing fatalities.

PDB data and structure‐guided approaches are widely used across the biopharmaceutical industry to support the discovery and development of new small‐molecule drugs (Burley 2021; Burley et al. 2024; Westbrook et al. 2020; Westbrook and Burley 2019). Below, we describe how visualizing and analyzing 3D biostructure information freely available from RCSB.org can help us understand how drugs act and how they can bind to other human proteins (potentially giving rise to unwanted off‐target‐related side effects). This information can help guide the discovery and development of next‐generation antiviral agents to reduce case‐fatality rates in the face of an H5N1 epidemic/pandemic.

The three classes of drugs currently used to treat influenza virus infections include NA inhibitors, adamantanes, and inhibitors of the RdRp cap‐dependent endonuclease activity. The US Food and Drug Administration (FDA) has approved two chemically similar NA inhibitors (zanamivir and oseltamivir—wwPDB CCD IDs ZMR and G39, respectively) that prevent the spread of infection with efficacy against a broad range of NA subtypes (Note: Oseltamivir is an oral pro‐drug, which is converted during passage through the liver into its active metabolite oseltamivir carboxylate). The US FDA‐approved drug amantadine (wwPDB CCD ID 308) blocks the M2 ion channel and interferes with viral entry into the host cell cytoplasm. Liabilities limiting its usage include toxic side effects and the emergence of resistance. The most recently approved influenza drug baloxavir marboxil is a prodrug rapidly converted to baloxavir acid (wwPDB CCD ID EwZ) by arylacetamide deacetylases occurring in cells of the blood, liver, and lumen of the small intestine.

**FIGURE 9:**
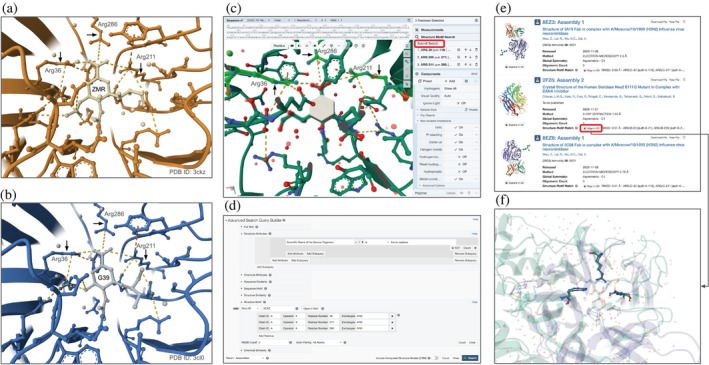
Launching a Structure Motif Search using Mol*. (a) Panel showing zanamivir binding to H5N1 NA (PDB ID 3ckz; Collins et al. 2008). Interacting amino acids within 5 Å are shown in ball‐and‐stick representation, with ionic interactions rendered as orange dashed lines. (b) Panel showing ionic interactions stabilizing oseltamivir binding (PDB ID 3cl0; Collins et al. 2008). (c) Selection of the Arg‐triad in PDB ID 3ckz for Structure Motif Search. (d) Advanced Search Query Builder set up to run the Structure Motif Search (specified in (c)) and refined to list only result structures that include at least one human protein. (e) Structure Motif Search results page listing human sialidase (NEU2). (f) Structure alignment of the Arg‐triad in both the H5N1 NA (green) and human NEU2 (violet).

Comparing MX co‐crystal structures of NA bound to zanamivir (PDB ID 3ckz) (Collins et al. 2008) and oseltamivir carboxylate (PDB ID 3cl0) (Collins et al. 2008) can reveal amino acids critical for the binding of both drugs. The Pairwise Structure Alignment Tool, described above, can be used to accomplish this objective. Visual inspection of the binding sites of both drugs reveals a triad of Arg residues (Arg 36 [auth 118], Arg 211 [auth 292], Arg 286 [auth 371]) that form analogous ionic interactions with zanamivir and with oseltamivir carboxylate (Figure 9a,b). Binding mode similarity suggests that it is worth enquiring whether a similar Arg‐triad exists in human and/or other viral proteins. Answering this question could help identify potential off‐target interactions with human proteins and/or identify additional viral targets for which one or both drugs may be effective.

The RCSB.org Structure Motif Search Tool (Bittrich et al. 2020) is ideally suited to this task. By visualizing the structure of NA bound to zanamivir (PDB ID 3ckz) (Collins et al. 2008) in Mol* (Figure 9c; hydrogen bonds have been hidden for the sake of clarity), each member of the Arg‐triad can be selected (in Selection mode) to define the search. At the time of writing, single‐mouse‐click of the Search button (Figure 9c) returned more than 775 experimentally determined PDB structures. Selecting the subset including at least one human protein narrows the result set to ~115 cases. Scrolling through the query results reveals Sialidase Neu2 (PDB ID 2f25) (Chavas et al. 2005). A representation of this refined query in the Advanced Search Query Builder (Figure 9d) can be saved for re‐running at a later date. See Figure S9 for details.

Single‐mouse‐click of the Align in 3D button on the results page (Figure 9e) delivers a comparison of the query structure (PDB ID 3ckz) and the Sialidase Neu2 search result (PDB ID 2f25) in a new Mol* window (Figure 9f). The RMSD for non‐hydrogen atom positions of the Arg‐triad is ~0.5 Å for PDB ID 3ckz versus PDB ID 2f25. In contrast, comparing the structures of the viral NA (PDB ID 3ckz) and the human sialidase (PDB ID 2f25) yields RMSD ~3.9 Å (sequence identity ~11%, 253 equivalent *α*‐carbon atomic pairs). While these two proteins appear somewhat distantly related in evolutionary terms, the Arg‐triad is common to both structures suggesting that zanamivir may bind to the active site of human NEU2.

In the clinic, neuropsychiatric adverse events and fatalities due to oseltamivir therapy have been reported (Fuyuno 2007; Maxwell 2007). A follow‐up study examining in vitro inhibitory effects of both oseltamivir carboxylate and zanamivir on select human sialidases (NEU1, NEU2, NEU3, and NEU4) documented that oseltamivir carboxylate does not bind to any of the four human enzymes (albeit the studies use recombinant preparations of enzyme) at 1 mM concentration, whereas zanamivir binds to NEU1, NEU2, NEU3, and NEU4 in the micromolar range (Hata et al. 2008). At the time of writing, repeating the Arg‐triad Structure Motif Search to include more than 1 million CSMs available at RCSB.org (by activating the CSM toggle switch visible in Figure 9d) returned 10 human protein CSMs—7 are sialidases (wherein the Arg‐triad matches with RMSD <1 Å) plus Transmembrane protein 132A, Breast cancer type 1 susceptibility protein, and Ubiquitin carboxyl‐terminal hydrolase 6 (all with higher RMSDs ~1.5–2 Å).

Oseltamivir treatment adverse events may be due to the binding of its active metabolite to one or more human sialidases not previously tested. Not surprisingly, the Arg‐triad Structure Motif Search also returned experimentally determined structures of other influenza virus NAs. As the genomes of new influenza viruses are isolated and sequenced and relevant NA structures are deposited into the PDB, RCSB.org tools described herein could be used to understand resistance to zanamivir and/or oseltamivir therapy (if/when it becomes clinically apparent) and provide structural guidance for the discovery/development of novel antiviral drugs based on chemically distinct chemotypes.

The PDB archive also houses co‐crystal structures of amantadine and baloxavir acid bound to their respective target proteins. PDB ID 3c9j (Stouffer et al. 2008) revealed at the atomic level the molecular mechanism of action of amantadine blocking an influenza A H5N1 M2 ion channel. Similarly, PDB ID 6fs6 (Omoto et al. 2018) revealed at the atomic level the molecular mechanism of action of baloxavir acid inhibiting influenza A H1N1 RdRp cap‐dependent endonuclease. Both of these structures can be explored using RCSB.org web portal tools as described for the NA inhibitors above.

Successful management of any zoonotic influenza A H5N1 virus pandemic/epidemic will require large‐scale vaccination. Viral subtypes known to circulate routinely in humans targeted by seasonal flu vaccines include A(H1N1), A(H3N2), and B(Victoria) (Centers for Disease Control and Prevention and National Center for Immunization and Respiratory Diseases (NCIRD) 2024). Their main immunogens are HA proteins (Nichol and Treanor 2006). Because influenza virus RdRps are error‐prone, viral genomes undergo continuous evolution potentially rendering seasonal vaccines ineffective against new subtypes (including H5N1). At the time of writing, the globally predominant H5N1 clade is 2.3.4.4b (Li et al. 2020). Several inactivated or attenuated vaccines against influenza A H5N1 viruses have been tested in animal models (Cargnin Faccin and Perez 2024).

3D structures of H5N1 HA proteins and those bound to antibody Fab fragments (e.g., PDB IDs 3fku (Sui et al. 2009), 4fqi (Dreyfus et al. 2012), and 8txt (McIntire et al. 2024)) archived in the PDB are freely available to help guide vaccine designers (Goodsell and Burley 2022). Recently, a nucleoside‐modified mRNA vaccine encoding clade 2.3.4.4b HA was tested in animal models (Furey et al. 2024). Experience with structure‐guided design, development, and regulatory approval of mRNA vaccines against SARS‐CoV‐2 (Burley 2025; Corbett et al. 2020) bodes well for the successful management of possible zoonotic H5N1 influenza virus epidemics (or global pandemic).

## 3 SUMMARY OF EXPLORATIONS USING RCSB.org Mol*

Exploration of influenza A H5N1 virus proteins (Figure 1) and their complexes with one another and small‐molecule drugs has demonstrated various ways to use RCSB.org Mol* and its deployments across RCSB PDB software tools.

Our explorations fell into three groups:

1. **Exploration of individual structures** to learn about overall shape, oligomeric assemblies and their symmetry, detailed structural features, and intermolecular interactions. PDB structures and CSMs can be visualized in various formats. wwPDB validation report information, experimental density maps, and structure–function annotations can be mapped onto 3D biostructures to learn more about structure quality and the biochemical and biological role(s) played by each protein (Figures 2, 3, 4, 5).
2. **Comparing structures** to understand conformational changes due to RNA binding and the impact of mutations/sequence variations on 3D structure. RCSB.org structure comparison features and our Pairwise Structure Alignment Tool support analyses of diverse structures (Figures 6, 7, and 9). In addition, groups of structures related by UniProt Accession Number (100% identity) or polymer sequence (lower sequence identity) can be viewed together and compared to learn about ligand binding and conformational flexibility (Figure 8).
3. **Identifying related structures** using the RCSB.org Structure Motif Search Tool can reveal common structural features of interest to both evolutionary biologists and drug hunters (Figure 9).

The RCSB.org Mol*‐based tools can support identification, exploration, examination, and comparison of structures relevant to almost any biological theme or topic of interest.

## 4 COMMUNICATING KNOWLEDGE AND SUPPORTING COLLABORATIONS

RCSB.org supports several ways to export and store Mol* visualizations. Users can bookmark or modify links from SSPs by changing relevant parameters. For preparation of publication‐ready figures, Mol* offers an image export option via the Screenshot/State menu (camera lens aperture icon) located in the top‐right corner of the Mol* 3D Canvas. This menu also supports the export of the current state or session file saving (bundling all source data and required when working with local files).

Three additional data export options are available at the bottom of the right‐hand Mol* panel:

1. Export models: Downloads atomic coordinates for PDB structures in PDBx/mmCIF or BinaryCIF formats.
2. Export animation: Creates short movies, such as a 360° rotation of a 3D structure in .mov file format.
3. Export geometry: Converts atomic coordinates into 3D shapes ready for 3D printing in .glb, .stl, .obj, or .usdz file formats.

## 5 CONCLUSION

The RCSB.org Mol* is a powerful, versatile, and easily accessible, no‐cost, web‐based molecular visualization tool. It can perform complex calculations and mappings onto 3D biostructure data and create publication‐quality images and animations. A wide range of features, use cases, and related tools are showcased herein with the proteome of the influenza A H5N1 virus, a potential threat to global public health. Other biologically and medically important topics and themes may be similarly explored using RCSB.org Mol*.

### 5.1 Opportunities for future enhancement for Mol*

Displaying sophisticated, customized scenes using Mol* can be challenging, particularly for more complex visualizations. PDBe and RCSB PDB are co‐developing the MolViewSpec standard, which allows users to define molecular visualizations and scenes in a declarative manner (Bittrich et al. 2024a). MolViewSpec provides a step‐wise process for composing human‐readable and human‐editable JSON files that combine small, intuitive building blocks. These files can be read and displayed by RCSB.org Mol*, consistently recreating previously defined scenes. Looking ahead, we aim to migrate aspects of RCSB.org Mol* to MolViewSpec, which will make scene definition and customization more transparent and more easily reusable, enhancing accessibility and collaboration across research and educational communities. Additional ongoing improvements to the core Mol* library include support for mesoscale visualizations encompassing billions of atoms or entire cells (molstar.org/me) (Rose et al. 2024).

## 6 MATERIALS AND METHODS

### 6.1 RCSB.org Mol* wrapper

A dedicated wrapper implements common Mol* usage patterns within the RCSB.org web portal. This custom wrapper enables branding and supports a range of additional features relevant to our research‐focused web portal. Related open‐source software projects, including the rcsb‐molstar wrapper, are enumerated in the “Accessibility Statement” section.

### 6.2 Standalone Mol*

All Mol* deployments across RCSB.org, except Standalone Mol*, are linked to individual PDB IDs or CSMs, or specific RCSB.org tools. Standalone Mol* is accessible at rcsb.org/3d-view, allowing the loading of multiple structures, including those from external sources. After loading the desired data, structure alignments can be computed directly in Mol* provided the biopolymer chains are similar in sequence.

### 6.3 Predicting the location of the phospholipid bilayer

The predict membrane tool (Bittrich et al. 2022) supports the prediction of the membrane bilayer location. It is available for any PDB structure annotated as a membrane protein by UniProt and represented in one or more trusted external membrane protein data resources (Dobson et al. 2024; Lomize et al. 2012; Newport et al. 2019; White 2009). Membrane placement (Postic et al. 2016) is based on amino acid residue solvent exposure and optimized for the orientation and thickness of the membrane bilayer such that superficial hydrophobic amino acid sidechains fall within the phospholipid bilayer while hydrophilic residues are solvent accessible.

### 6.4 Symmetry‐related visualization

Visual display of symmetry axes detected in a macromolecular assembly can be accessed from SSPs or requested in Mol* directly using the Assembly Symmetry panel. Pre‐calculated symmetry‐related data is fetched by the RCSB.org Data API at data.rcsb.org.

### 6.5 Static images and data preparation for the RCSB.org web portal

The Molrender Tool is used to generate static images in JPEG format. These images are displayed on SSPs for individual PDB structures or CSMs and serve as previews in search results. The core Mol* technology stack plays a crucial role in data preparation for weekly updates of the RCSB.org web portal. Command‐line tools are used to create optimized binary representations of PDBx/mmCIF source files in BinaryCIF format (Sehnal et al. 2020). These tools also preprocess experimental density map data from MX and 3DEM studies, facilitating the efficient delivery of volumetric data.

### 6.6 Viewing sequence annotations in 3D

The RCSB PDB Sequence Annotations in 3D Viewer (Segura et al. 2022) connect information about the 1D sequence to locations of key residues in the 3D view and vice versa. Users can hover over or single‐mouse‐click on individual residues in the 3D view (right) to highlight the corresponding position in the 1D sequence view (left). Conversely, doing the same with a position or feature in the 1D sequence view highlights its location in the 3D view (Figure 3b).

### 6.7 Pairwise structure alignments

3D structure alignments can be performed using the RCSB.org Pairwise Structure Alignment Tool (rcsb.org/alignment) (Bittrich et al. 2024b). This user‐friendly feature supports alignment of up to nine chains versus a single reference chain with well‐established alignment algorithms (i.e., TM‐align (Zhang and Skolnick 2005) or jFATCAT, jCE, jCE‐CP, Smith‐Waterman 3D from the BioJava library (Lafita et al. 2019)). Results are presented interactively, allowing users to explore alignments at both 1D sequence and 3D structure levels. Sequence alignments are visualized using the Sequence Annotations in 3D Viewer (Segura et al. 2022), which summarizes structurally aligned regions, sequence‐only matches, and unaligned regions or gaps. In parallel, Mol* provides a dynamic 3D structure view of aligned polypeptide chains. Alignments can be readily interrogated as selections made in the 1D sequence view are automatically reflected in the 3D structure view and vice versa.

### 6.8 Comparing 1D sequence and 3D structure alignments

RCSB PDB Group Summary Pages (Segura et al. 2023) allow users to explore related entities (e.g., those with a common UniProt Accession Number). This web page presents an interactive side‐by‐side view, similar to the Pairwise Structure Alignment Tool (described below). Panels on the left of Figure 8c,e display the 1D sequence alignment of all corresponding entities, together with shared key UniProt annotations. Entities are sorted in descending order based on the extent of sequence coverage concerning the full‐length polypeptide chain. Unresolved portions of polypeptide chains for experimentally determined structures in the PDB are indicated by gray sections of the horizontal track. CSM horizontal tracks are colored according to pLDDT confidence scores (Tunyasuvunakool et al. 2021). Initially, the first entity appearing in the alignment list is displayed in 3D (right). Additional entities in the 1D sequence alignment can be displayed in the 3D view by clicking on their desaturated identifier labels in the left‐hand panel. For finer control over displayed entities, the three square boxes can be used to gain an overview of entity composition and toggle 3D representations of the aligned entity, other polymeric chains, or bound ligands (as applicable). This approach to displaying available structure information allows users to quickly assess the relevance of individual PDB IDs or CSMs to the scientific question(s) at hand.

### 6.9 Structure motif searching of 3D structures

The RCSB.org web portal offers a dedicated Structure Motif Search Tool (Bittrich et al. 2020), which can rapidly identify similar 3D arrangements of selected amino acids in PDB structures or CSMs. This tool harnesses Mol* to streamline the construction of queries and interrogation of results as 3D structure alignments. Users can also define Structure Motif queries using the RCSB.org Advanced Query Builder by specifying a reference structure and selecting from 2 to 10 residues by specifying chain identifiers and sequence positions. Because this process can be tedious and requires knowledge of exact sequence positions for residues of interest, RCSB.org Mol* provides a dedicated user interface allowing users to define structure motifs interactively in 3D.

### 6.10 Accessibility statement

The RCSB.org web portal is freely accessible and does not require user registration. All software dependencies and the entire Mol* technology stack are shared via GitHub (github.com/molstar). General‐purpose functionality is part of the core Mol* project (github.com/molstar/molstar, landing page: molstar.org, available as npm download: npmjs.com/package/molstar). Custom functionality specific to RCSB.org is implemented in a dedicated RCSB.org Mol* wrapper (github.com/molstar/rcsb-molstar, available as npm download: npmjs.com/package/@rcsb/rcsb-molstar). The Sequence Annotations Viewer software stack is available at: github.com/rcsb/rcsb-saguaro, github.com/rcsb/rcsb-saguaro-app, and github.com/rcsb/rcsb-saguaro-3d. The user interface of the Pairwise Structure Alignment Tool is shared at: github.com/rcsb/rcsb-pecos-app. Static images can be generated at scale using the Mol* Molrender Tool (github.com/molstar/molrender). Ongoing joint progress by PDBe and RCSB PDB on the MolViewSpec standard is also freely available (github.com/molstar/mol-view-spec, landing page: molstar.org/mol-view-spec, PyPI download: pypi.org/project/molviewspec). Mol* makes direct use of the RCSB PDB Data API (data.rcsb.org) (e.g., to retrieve data for the visualization of protein symmetry). Note: PDBe maintains its custom wrapper at github.com/molstar/pdbe-molstar.

## AUTHOR CONTRIBUTIONS

**Sebastian Bittrich:** Writing – review and editing; writing – original draft; visualization; software. **Alexander S. Rose:** Conceptualization; project administration; visualization; software; supervision. **David Sehnal:** Supervision; project administration; conceptualization; funding acquisition; software; visualization. **Jose M. Duarte:** Conceptualization; project administration; software; visualization; supervision; writing – review and editing. **Yana Rose:** Conceptualization; visualization; project administration; supervision; software. **Joan Segura:** Supervision; software; visualization. **Dennis W. Piehl:** Visualization; writing – review and editing; software. **Brinda Vallat:** Software; visualization; writing – review and editing. **Chenghua Shao:** Software; validation; visualization; writing – review and editing. **Charmi Bhikadiya:** Software; visualization. **Jesse Liang:** Software; visualization. **Mark Ma:** Software; visualization. **David S. Goodsell:** Visualization; writing – original draft; writing – review and editing. **Stephen K. Burley:** Conceptualization; writing – original draft; writing – review and editing; funding acquisition; supervision. **Shuchismita Dutta:** Conceptualization; writing – original draft; writing – review and editing; supervision.

## CONFLICT OF INTEREST STATEMENT

The authors declare no conflicts of interest.

## Supporting information


**Data S1.** Supporting Information.

## Data Availability

Data sharing is not applicable to this article as no new data were created or analyzed in this study.
