# Supplementary material for: Visualizing and analyzing 3D biomolecular structures using Mol* at RCSB.org: Influenza A H5N1 virus proteome case study
Source: Protein Sci. 2025 Mar 18;34(4):e70093. doi: 10.1002/pro.70093 (PMC11915458; doi:10.1002/pro.70093)
Supplement: Supplementary file 1 — Data S1. Supporting Information. [file PRO-34-e70093-s001.pdf]

Supplementary Materials for RCSB.org Mol*, a molecular visualization tool

This supplementary material provides instructions for recreating most of the visualizations from the main manuscript, along with additional details where needed. The document is organized by individual figures and mirrors the structure of the main text. Step-by-step instructions show how users can use RCSB.org Mol* to reproduce the visualizations in the context of the biological narrative.

Figure 1: Illustrative Rendering of the H5N1 Virion

To create images in the illustrative style shown in Figure 1 (right), the representation, lighting, outlines, and projection mode need to be adjusted. Use the following steps:

0. **Open the File of Interest in Mol***: e.g., 2fk0**.**
1. **Change to Spacefill Representation:**
    a. Hide existing Polymer and Carbohydrate representations by clicking on the respective eye icons.
    b. Click the three-dot icon on the right of the Polymer component to access more options.
    c. Click Add Representation, scroll down to the Spacefill option, and select it. You can now see all atoms of the structure drawn as spheres.

2a. **Apply Uniform Coloring:**
    a. Click on the newly created Spacefill representation dropdown at the bottom to expand it and see more options.
    b. Click on the name of the Color Theme and change it from Element Symbol to Uniform (in the Miscellaneous group). The structure is now colored uniformly in gray.

2b. **Set to Desired Color**:
    a. Click on the three-dot icon to access advanced options and click on the gray area labeled “Value” to change the color from gray to an RGB value of (169, 75, 255). This changes everything to the chosen color.

3. **Apply Simplified Shading**:
    a. For the Spacefill representation, click on the three-dot icon to the right of the Spacefill type, navigate to Advanced Options and then Shading.
    b. Toggle the Ignore Light option to On.
4. **Adjust Projection Mode and Outlines:**
    a. In the vertical menu shown on the 3D Canvas, click on the Settings/Control button.
    b. In the popup box, set the Camera (projection) to Orthographic to ensure all atoms are sized consistently without perspective distortion.
    c. In the same popup box, turn on the Outlines option.

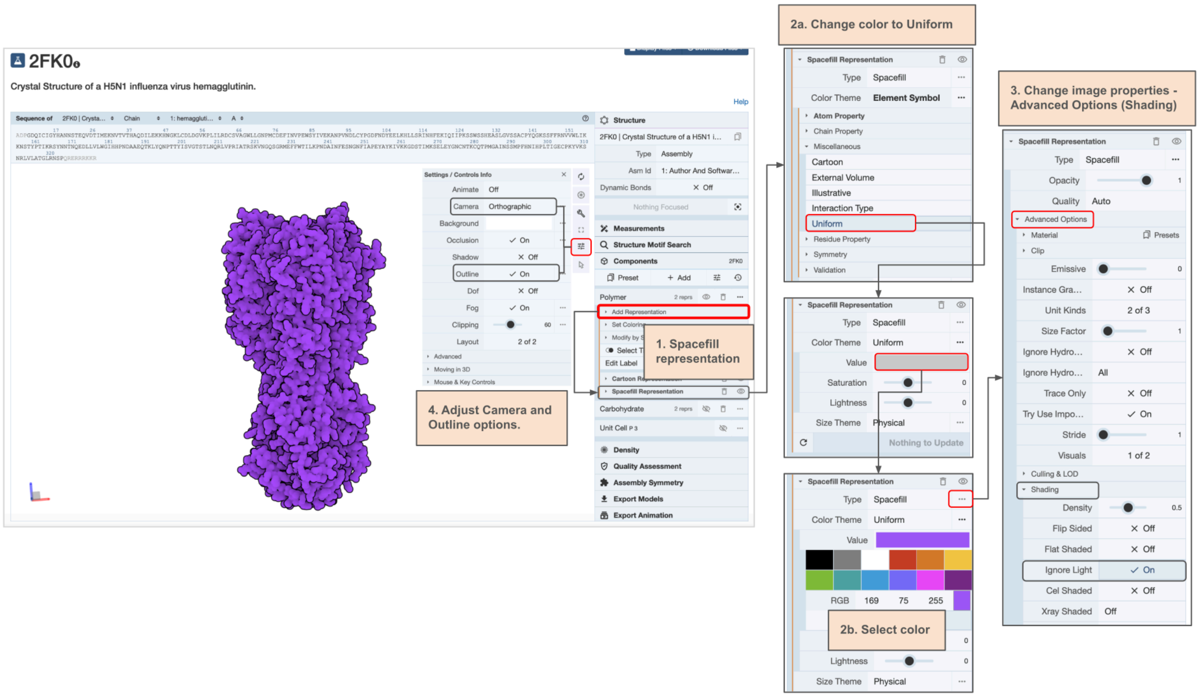

Figure S1: Steps for creating the rendition of 3D structures shown in Figure 1 right. Step 1 sets the representation to spacefill. Step 2 applies a uniform color. Step 3 adjusts the lighting. Step 4 adds outlines and changes the projection mode.

Figure 2: Surface Representation for H5N1 Hemagglutinin Protein

To create images shown in Figure 2D, use the following steps:

1. **Open PDB ID 2fk0 in Mol*.**
2. **The Mol* Interface:**
   The Mol* tool's user interface has three distinct sections (see Figure 2B and S2) – (i) Sequence panel, which displays the polymer sequence and lists any small molecule ligand entities present in the structure; (ii) the 3D Canvas, which displays the 3D structure interactively; and the (iii) the Controls panel, which allows various operations related to the molecular visualization.
3. **Activate Selection Mode**: Click the mouse cursor icon on the 3D canvas (right-most red emphasis in Supplementary Figure S2). This will bring up the menu with Selection operations.
4. **Change Selection Granularity to Chain:** Use the dropdown menu on the left to change the selection granularity (*e.g.*, individual atoms, residues, …) from the default Residue value to Chain to select complete chains with a single click.
5. **Select Chain E**: Click on the purple chain, oriented toward the top-right corner. Use the tooltip that appears to confirm that you selected chain E.
6. **Create Component and Representation**:
   a. Create a dedicated component describing chain E by clicking the cube icon in the Selection operations menu.
   b. Keep Selection as Current Selection.

c. Specify Molecular Surface as Representation.
d. Click “+ Create Component” to apply this selection and create an additional surface representation for chain E.

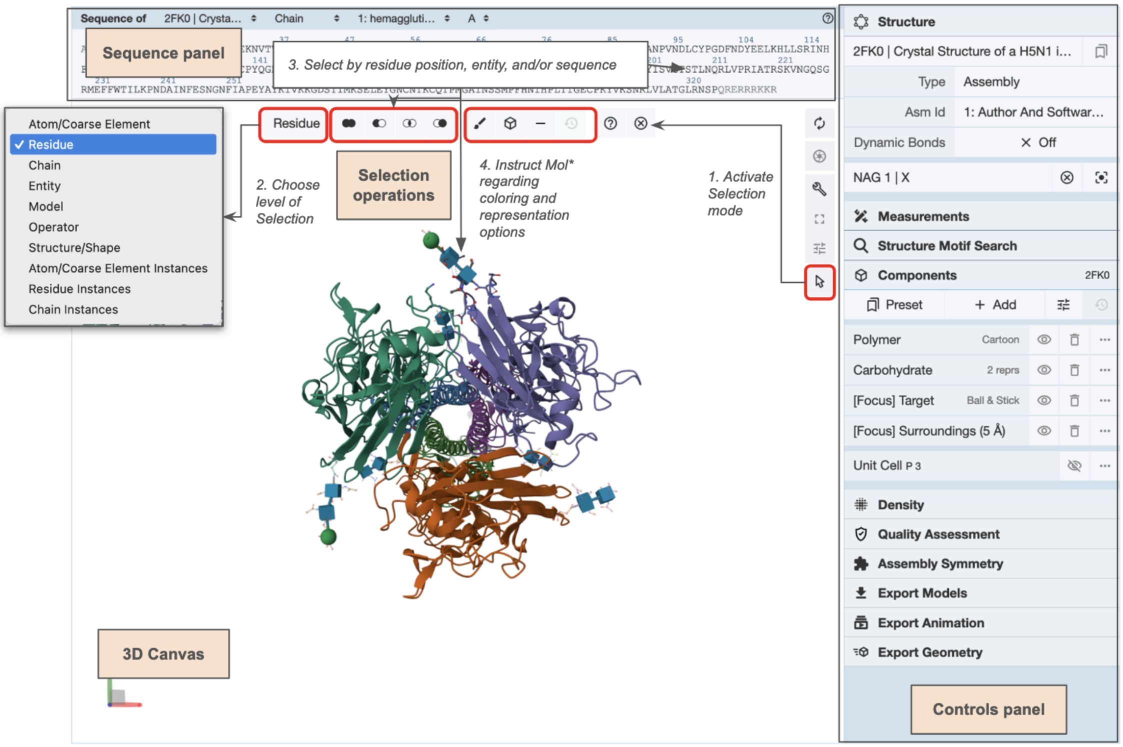

Figure S2: Interface of RCSB.org Mol* showing the 3D Canvas, Sequence panel, and Controls panel. The structure displayed is for PDB ID 2fk0. Toggle the Selection Mode using the mouse cursor icon (right-most red box) to show Selection options in the horizontal panel of options at the top of the 3D Canvas.

Figure 3: RSCC Coloring for H5N1 Hemagglutinin Protein

To color an MX structure by real-space correlation coefficient (RSCC) similar to Figure 3E, use the following steps:

1. **Open PDB ID 2fk0 in Mol*.**
2. **Click on the Quality Assessment Section in the Controls Panel.**
3. **Select Experimental Support Confidence Option**: Note this is only available for structures determined by MX.
4. **Hover over Individual Residues to See Details**: Numerical RSCC values will be shown in the tooltip at the bottom-right corner of the canvas.

Note that this color scheme used is similar to pLDDT confidence scores, with reliable regions shown in blue and problematic regions in yellow or orange (Shao et al., 2022).

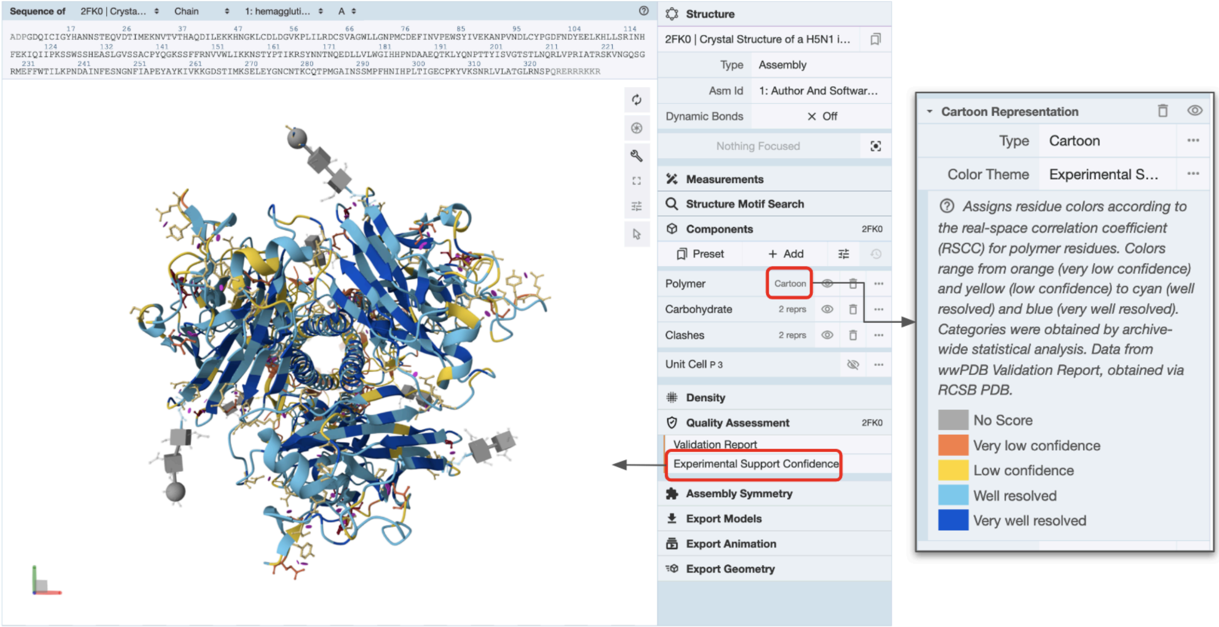

Figure S3: The structure of PDB ID 2fk0 colored by RSCC values.

Figure 4: Non-Covalent Interactions for H5N1 Neuraminidase Inhibited by Zanamivir

Examining covalent and non-covalent interactions in the vicinity of a specific residue or ligand can provide insights about how it is stabilized and/or can play a role in the molecule's function. There are multiple ways in which you can display these interactions as seen in Figure 4B.

1. **Load a Structure and Click on Residue or Ligand of Interest in the Default Mode of Mol***: This zooms in and shows environment and non-covalent interactions within a 5 Å radius. Note that this function is disabled in Selection Mode.
2. **To Include or Exclude Specific Interaction Types**:
    a. **Expand the Components Panel**.
    b. **Expand Global Representation Options**. Do so by clicking the third button with the slider icon (step 1 in the Figure S4).
    c. **Expand the Non-covalent Interactions Section** (step 2 in the Figure S4).
    d. **Toggle Types as Desired** (step 3 in the Figure S4). The supported types are:
        - Ionic interactions
        - π-stacking interactions
        - π-cation interactions
        - Halogen bonds
        - Hydrogen bonds
        - Weak hydrogen bonds
        - Hydrophobic interactions
        - Metal coordination

e. Use the right-most icon (three-dot icon) to access options specific to each interaction type. This allows you to customize interaction criteria and visuals (step 4 in the Figure S4).

3. **Activate the Selection Mode and Select Specific Amino Acids of Interest** (preferably, focus on amino acids near a ligand of interest or those mentioned in the literature).

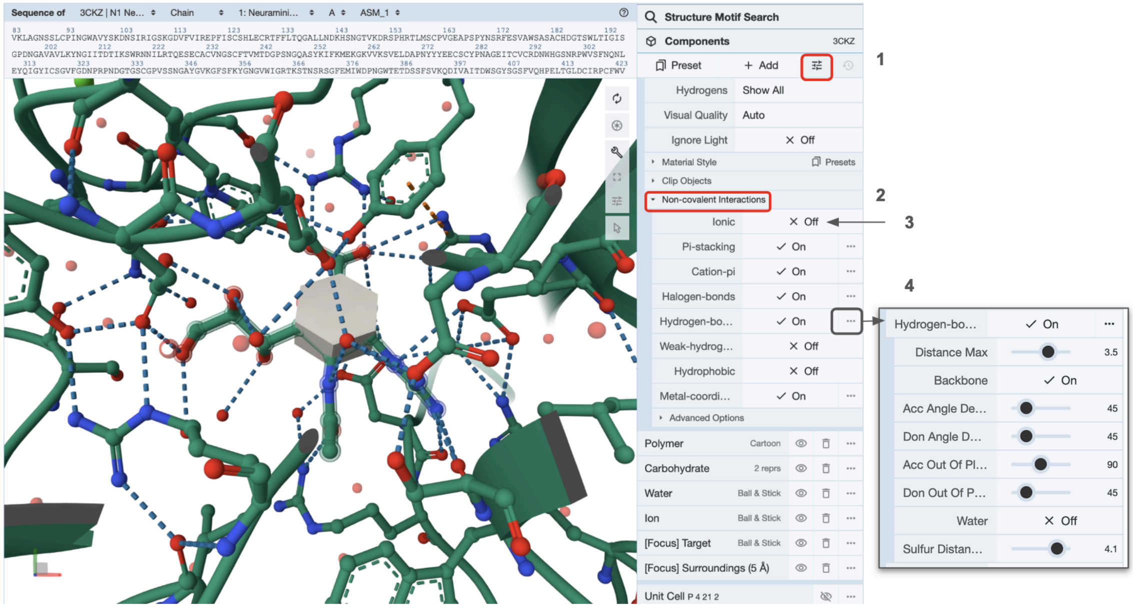

Figure S4: Customizing non-covalent interaction for PDB ID 3ckz.

Figure 5: Highlight & Measure Key Residues of the H5N1 M2 Ion Channel Pore

Recreate Figure 5D by following these steps:

1. **Open PDB ID 6nv1 in Mol***.
2. **Simplify View**. Use the Components panel to display the detected types of molecules and atoms. Click the trash bin icon for all components except the Polymer component to ensure only the four protein chains are rendered.
3. **Activate Selection Mode**. Refer to the instructions above on how to activate Selection Mode.
4. **Identify All His17 [auth 37] Residues**.
    a. Click on His17 [auth 37]. The sequence panel is helpful, especially for larger chains.
    b. Identify analogous instances of His17 [auth 37] in the other chains. You can do this visually or by repeating the previous step after switching the sequence panel content from chain A to other chains using the right-most dropdown menu.
5. **Identify All Trp21 [auth 41] Residues**.
    a. Click on Trp21 [auth 41]. Again, the sequence panel helps with larger chains.

b. Identify analogous instances of Trp21 [auth 41] in other chains. Either do this visually or by performing the previous step again after switching the sequence panel content from chain A to other chains.

6. **Add Ball & Stick Representation**. Follow the earlier instructions to create a new component with a Ball & Stick representation that captures all eight residues (4 His, 4 Trp).
7. **Perform Measurements**.
   a. Expand the Measurements panel by clicking on it.
   b. Ensure that Selection Mode is still active.
   c. Click on one of the His residues in Ball & Stick representation.
   d. Click on one of the two other proximal His residues.
   e. Click the option stating "Distance (top 2 selection items)" to calculate the distance and add the corresponding visuals.
   f. Repeat as needed for other residue pairs, after clearing the current selection by clicking the canvas.

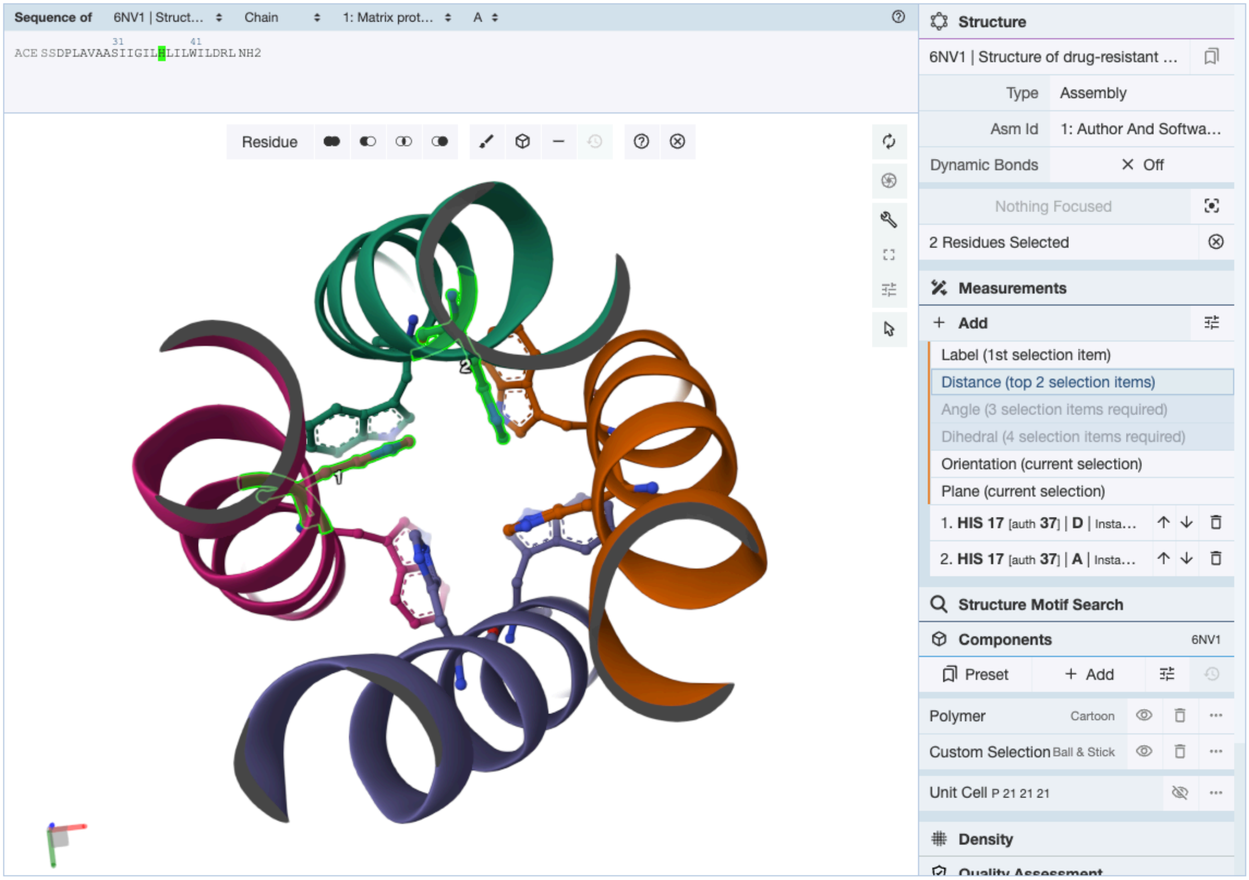

Figure S5: Performing measurements on PDB ID 6nv1.

Figure 6: Interrogating Conformational Changes in NP Upon RNA Binding Using the Standalone Viewer

Recreate the panels of Figure 6A by aligning chains directly in Mol* by following these steps:

1. **Open the Desired PDB IDs (2q06 and 7dxp) in Mol*.**
2. **Simplify View.** Create dedicated components for chain A of PDB ID 2q06 and chain B of PDB ID 7dxp, and hide or remove other components.
    a. **Activate Selection Mode**. Refer to the instructions provided above on activating Selection Mode.
    b. **Change Selection Granularity from Residue to Chain.** Use the menu that appears to adjust the selection granularity.
3. **Select Chains to Align.** Click on both chains. Mol* should report "2 Chains Selected."
4. **Superpose Both Chains.** Navigate through the Superposition menu: Chains > Superpose. If the structures share a UniProt accession number, you can also use the UniProt option to superimpose entries based on the alignment reported by UniProt.

The resulting root mean square deviation (RMSD) will be displayed in the Mol* console.

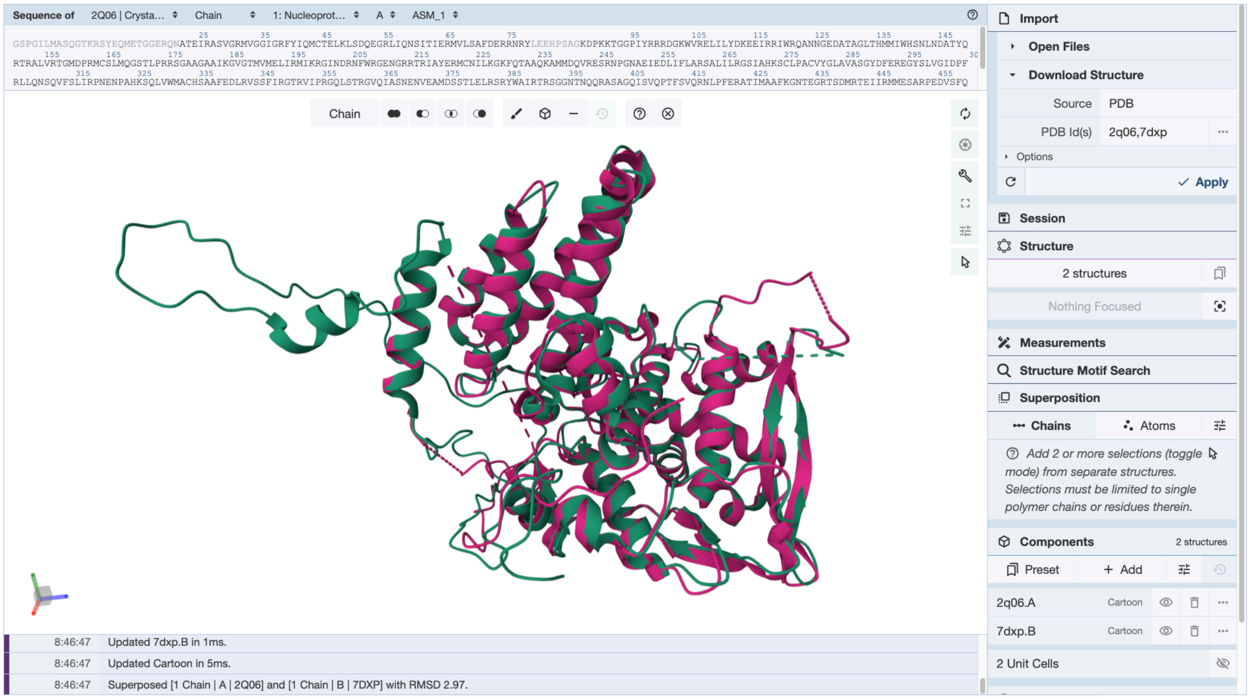

Figure S6: A pairwise alignment of PDB IDs 2q06.A and 7dxp.B.

Figure 7: Comparing Avian and Human-Specific HA using the RCSB.org Pairwise Structure Alignment Application

The Pairwise Structure Alignment application (rcsb.org/alignment) supports various input options. Chains can be selected from the PDB archive using either PDB IDs or UniProt Accession Numbers. Additionally, external data can be integrated by referencing identifiers from AlphaFold DB (Varadi et al., 2024) or ESM Metagenomic Atlas (Lin et al., 2023), or by providing a publicly accessible URL. External data from any source can be referenced using the File URL option if the macromolecular data is publicly available (make sure to specify PDBx/mmCIF or PDB format accordingly). Local files can also be uploaded using the File Upload option, where they will be converted to BinaryCIF and uploaded to the RCSB.org web portal. The alignment

application will then reference the uploaded file using the File URL option, allowing the alignment results to be bookmarkable and shareable *via* URL when local files are used as input.

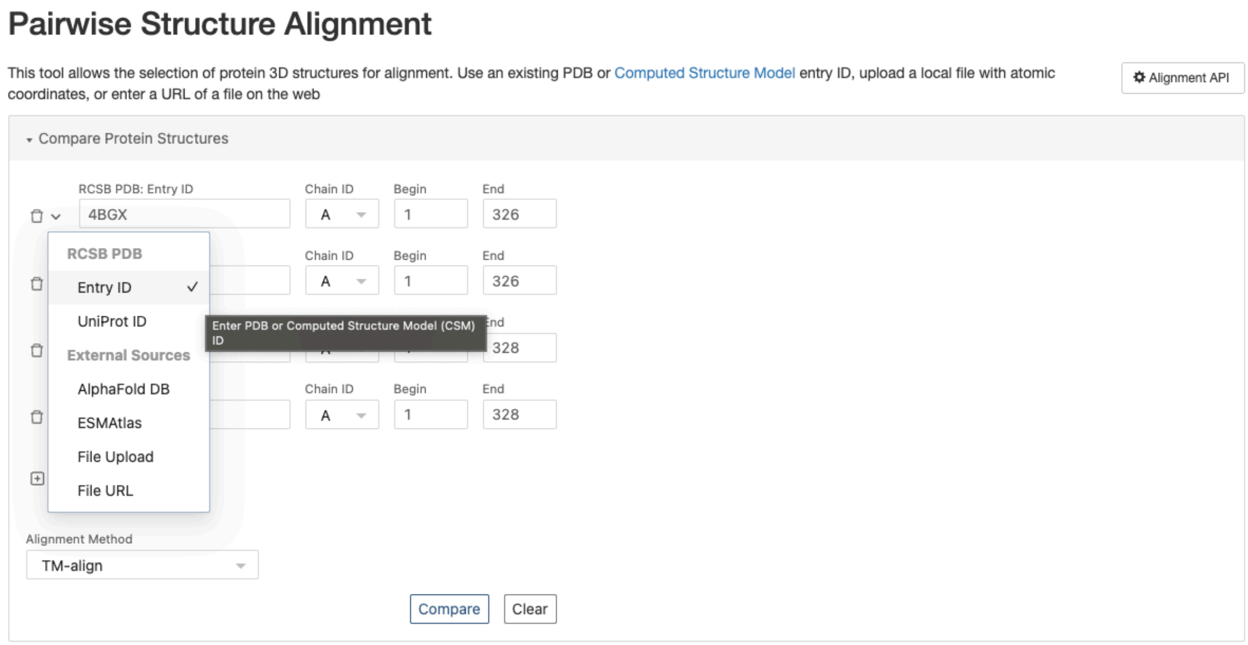

Figure S7: Input options supported by the Pairwise Structure Alignment application.

Alignment results can be downloaded in PDBx/mmCIF format, with all coordinates transformed according to the alignment. Additionally, alignments can be bookmarked and shared *via* a unique URL.

Figure 8: Create a Transparent Surface Representation for H5N1 Influenza A Polymerase Dimer in Complex With Human ANP32B

Panel 8A can be created by these steps:

1. **Load PDB ID 8r1j in Mol***.
2. **Activate Selection Mode**. Refer to the instructions provided earlier on activating Selection Mode.
3. **Switch to Chain Granularity**. Change the selection granularity to Chain.
4. **Select Chain G**.
5. **Invert Selection to Select Everything but Chain G**.
    a. Click the fourth icon for set operations (two circles, right one filled in black).
    b. Click Manipulate Selection to expand.
    c. Choose the Inverse / Complement of Selection option.
6. **Create Molecular Surface Representation**. This might take a moment.
7. **Lower Opacity of Newly Created Representation**.
    a. Click the three-dot icon of the Custom Selection component.
    b. Expand Molecular Surface Representation panel.

c. Click the three-dot icon of the Type property.
d. Reduce its opacity using the slider, *e.g.* down to 0.2.

8. **Activate Outline**. You can find this option as a global setting by using the slider icon in the vertical menu in the top-right corner of the canvas.

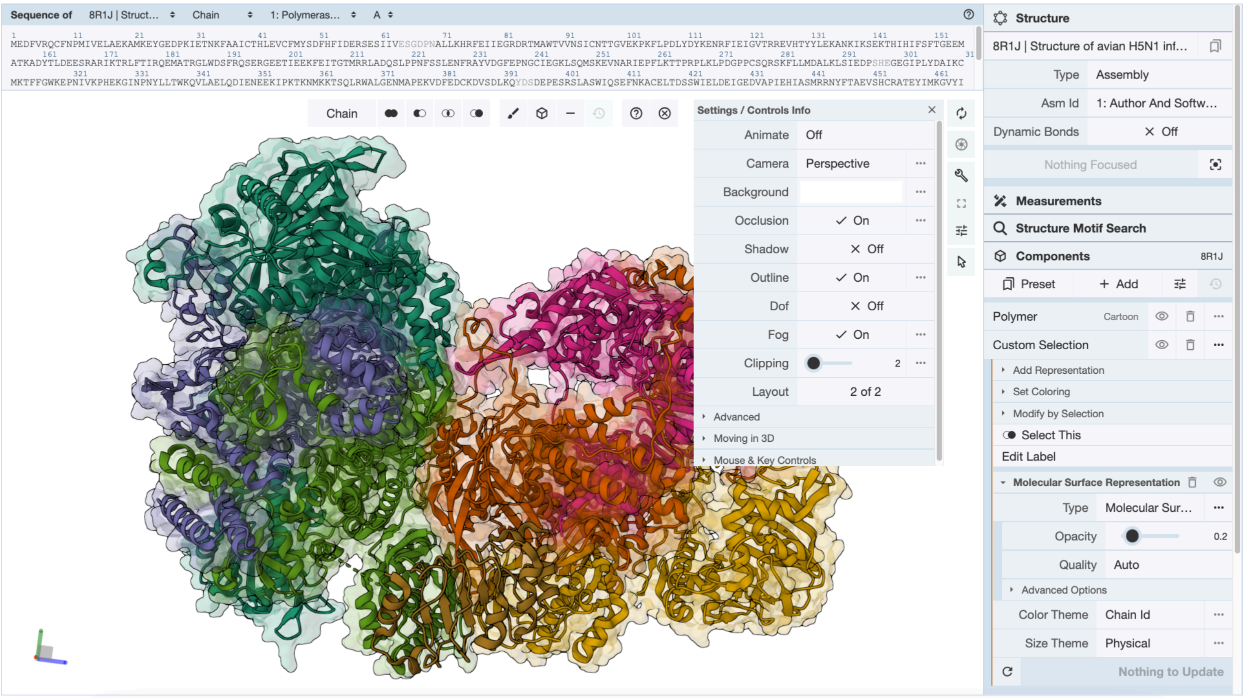

Figure S8: Creating a transparent surface representation.

Figure 9: Defining a Structure Motif Query Based on the Zanamivir Binding Site of H5N1 Neuraminidase

To define a Structure Motif query, use the RCSB.org Advanced Query Builder, specifying a reference structure and selecting 2 to 10 residues by chain identifiers and sequence positions. This process can be simplified by using the RCSB.org Mol* viewer's dedicated 3D interface for defining structure motifs. Literature and resources like the Mechanism and Catalytic Site Atlas (Ribeiro et al., 2018) provide well-studied motif definitions.

For PDB ID 3ckz, the following three-residue motif of arginines (Figure S7) yielded interesting results: Arg 36 [auth 118], Arg 211 [auth 292], and Arg 286 [auth 371]:

1. **Activate Selection Mode.** Refer to the instructions provided earlier on activating Selection Mode.
2. **Select Residues**. Click on these positions on the canvas or in the Sequence Viewer.
3. **Remove Residues**. Click the trash bin icon (as needed).

By default, the Structure Motif tool maintains the amino acid identity (*e.g.*, Arg36 [auth 118] will only match other arginines). You can allow up to three position-specific exchanges per residue

by toggling the corresponding amino acid/nucleotide codes using the provided list (after clicking the first slider icon).

Note: The RCSB.org website and its APIs use label_asym_id and label_seq_id to identify chains and sequence positions. For author-assigned sequence IDs (auth_seq_id), the Mol* Sequence Viewer should be used. Both identifiers will be shown in the bottom-right corner of the Mol* canvas when hovering over a residue.

To launch the search, click Submit Search, which populates the Advanced Query Builder with your inputs. The search results will list matched assemblies, ranked by similarity based on RMSD values.

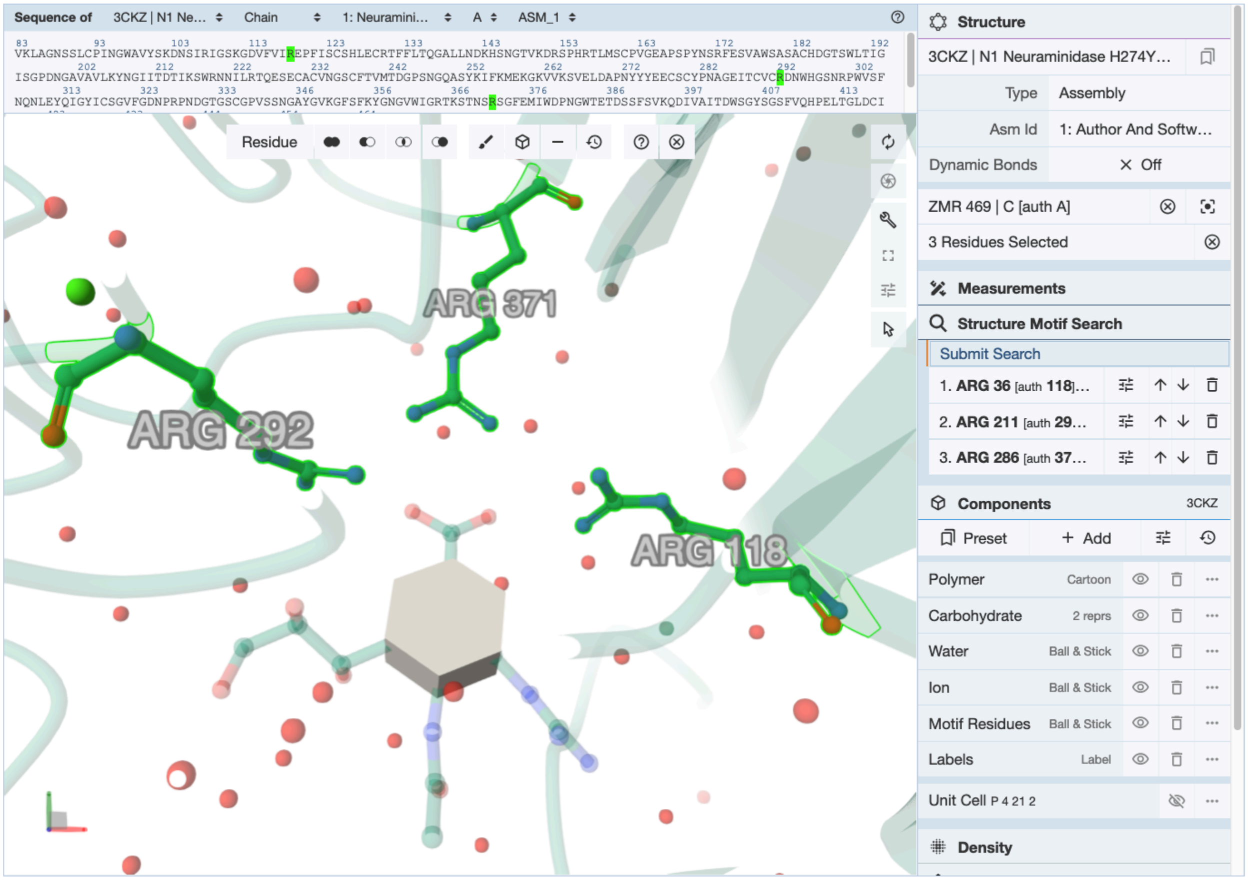

Figure S9: 3-residue motif definition for PDB ID 3ckz. Selected residues with green halo. Click Submit Search to run the query. Opacity reduced and labels added for visual clarity.

The Refinements panel left of the search results helps with navigating results (*e.g.*, most matches may be human proteins with cyclic symmetry). Individual motif alignments can be visualized in Mol* using the Align in 3D button, which opens the matched entries and creates ball-and-stick representations of motif residues. For instance, PDB ID 2bat (Varghese et al., 1992) has similar motif residues, with its sialic acid ligand placed analogously to Zanamivir in

PDB ID 3ckz. The RMSD value between motif residues and the matched sequence positions is displayed next to the visualization link.

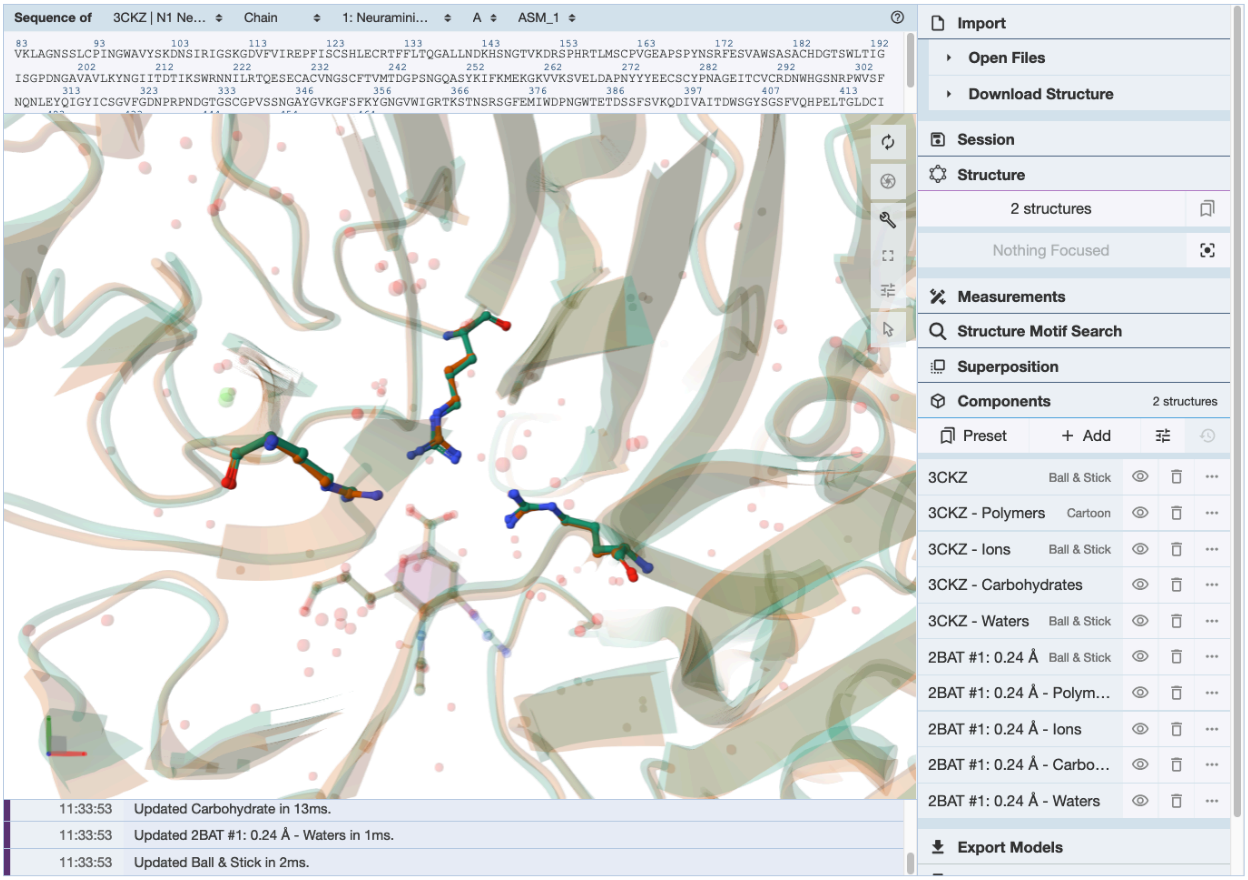

Figure S10: Motif alignment for PDB IDs 3ckz (colored green) and 2bat (colored orange). Open by following this link. Matched motifs, ligand placement, and overall structure match closely.

Additional References

Lin Z, Akin H, Rao R, Hie B, Zhu Z, Lu W, Smetanin N, Verkuil R, Kabeli O, Shmueli Y, et al. (2023) Evolutionary-scale prediction of atomic-level protein structure with a language model. Science 379:1123–1130.

Ribeiro AJM, Holliday GL, Furnham N, Tyzack JD, Ferris K, Thornton JM (2018) Mechanism and Catalytic Site Atlas (M-CSA): a database of enzyme reaction mechanisms and active sites. Nucleic Acids Research 46:D618–D623.

Varghese JN, McKimm-Breschkin JL, Caldwell JB, Kortt AA, Colman PM (1992) The structure of the complex between influenza virus neuraminidase and sialic acid, the viral receptor. Proteins : Structure, Function, and Genetics. 14(3):327-332.
